# Association between the XRCC1 Arg399Gln Polymorphism and Risk of Cancer: Evidence from 297 Case–Control Studies

**DOI:** 10.1371/journal.pone.0078071

**Published:** 2013-10-29

**Authors:** Liu Yi, He Xiao-feng, Lu Yun-tao, Long Hao, Song Ye, Qi Song-tao

**Affiliations:** 1 Neurosurgery Department, Nanfang Hospital of Southern Medical University, Guangzhou, PR China; 2 Department of Research, Peace Hospital of Changzhi Medical College, Changzhi, PR China; Sanjay Gandhi Medical Institute, India

## Abstract

**Background:**

The Arg399Gln polymorphism in the X-ray cross-complementing group 1 (XRCC1) had been implicated in cancer susceptibility. The previous published data on the association between XRCC1 Arg399Gln polymorphism and cancer risk remained controversial.

**Methodology/Principal Findings:**

To derive a more precise estimation of the association between the XRCC1 Arg399Gln polymorphism and overall cancer risk, we performed a meta-analysis of 297 case-control studies, in which a total of 93,941 cases and 121,480 controls were included. Overall, significantly increased cancer risk was observed in any genetic model (dominant model: odds ration [OR] = 1.04, 95% confidence interval [CI] = 1.01–1.07; recessive model: OR = 1.08, 95% CI = 1.03–1.13; additive model: OR = 1.09, 95% CI = 1.04–1.14) when all eligible studies were pooled into the meta-analysis. In further stratified and sensitivity analyses, significantly elevated hepatocellular and breast cancers risk were observed in Asians (dominant model: OR = 1.39, 95% CI = 1.06–1.84) and in Indians (dominant model: OR = 1.64, 95% CI = 1.31–2.04; recessive model: OR = 1.94, 95% CI = 1.09–3.47; additive model: OR = 2.06, 95% CI = 1.50–2.84), respectively.

**Conclusions/Significance:**

This meta-analysis suggests the participation of XRCC1 Arg399Gln is a genetic susceptibility for hepatocellular cancer in Asians and breast cancer in Indians. Moreover, our work also points out the importance of new studies for Arg399Gln association in some cancer types, such as glioma, gastric cancer, and oral cancer, where at least some of the covariates responsible for heterogeneity could be controlled, to obtain a more conclusive understanding about the function of the XRCC1 Arg399Gln polymorphism in cancer development.

## Introduction

DNA repair systems play critical roles in protecting against mutations and are essential for maintaining the integrity of the genome. Certain common genetic polymorphisms within the genes involved in DNA damage responses may contribute to the development of cancer and be associated with an increased risk of the disease. Because reduced DNA repair capacity may lead to genetic instability and carcinogenesis, genes involved in DNA repair had been proposed as candidate cancer susceptibility genes [1]. Until now, more than a hundred proteins implicated in DNA repair have been found in human cells. These proteins were implicated in four major DNA repair pathways, including nucleotide excision repair (NER), base excision repair (BER), double-strand break repair (DSBR) and mismatch repair (MMR) [1], [2].

The XRCC (X-Ray cross-complementing) genes were initially discovered through their role in DNA damage response caused by ionizing radiation. They are important components of various DNA repair pathways contributing to DNA-damage processing and genetic stability [3]. The DNA repair enzymes XRCC1 play a central role in the BER pathway [4], [5]. XRCC1 is located on chromosome no. 19q13.2–13.3, and its gene product is implicated in single-strand break repair and base excision repair mechanisms [6]. Although there are more than 300 validated single nucleotide polymorphisms (SNPs) in the XRCC1 gene reported in the dbSNP database (http://www.ncbi. nlm.nih.gov/SNP), three of which are common [7] and lead to amino acid substitutions in XRCC1 at codon 194 (exon 6, base C to T, amino acid Arg to Trp, dbSNP no. rs1799782), codon 280 (exon 9, base G to A, amino acid Arg to His, dbSNP no. rs25489) and codon 399 (exon 10, base G to A, amino acid Arg to Gln, dbSNP no.rs25487), these non-conservative amino acid changes may alter XRCC1 function. This change in protein biochemistry leads to the supposition that variant alleles may diminish repair kinetics, thereby influencing susceptibility to adverse health effects, including cancer.

In the past decade, a number of molecular epidemiological studies have been done to evaluate the association between XRCC1 Arg399Gln polymorphism and different types of cancer risk in diverse populations. However, the results were inconsistent or even contradictory. Partially because of the possible small effect of the polymorphism on cancer risk and the relatively small sample size in each of published studies. In addition, some recent meta-analyses analyzed such an association only for single cancer such as lung cancer, gastric cancer, cervical cancer, breast cancer, prostate cancer, and so on [8]–[12]. Therefore, we performed a comprehensive meta-analysis by including the most recent and relevant articles to identify statistical evidence of the association between XRCC1 Arg399Gln polymorphism and risk of all cancers that have been investigated. Meta-analysis is a powerful tool for summarizing the different studies. It can not only overcome the problem of small size and inadequate statistical power of genetic studies of complex traits, but also can provide more reliable results than a single case–control study.

## Materials and Methods

### Identification and Eligibility of Relevant Studies

A comprehensive literature search was performed using the PubMed, ISI, and EMBASE database for relevant articles published (the last search update was Jan. 15, 2013) with the following key words “XRCC1,” “polymorphism,” and “cancer” or “carcinoma.” The search was not limited to language. Additional studies were identified by hand searching references in original articles and review articles. Authors were contacted directly regarding crucial data not reported in original articles. In addition, studies were identified by a manual search of the reference lists of reviews and retrieved studies. We included all the case–control studies and cohort studies that investigated the association between XRCC1 Arg399Gln polymorphism and cancer risk with genotyping data. All eligible studies were retrieved, and their bibliographies were checked for other relevant publications. When the same sample was used in several publications, only the most complete information was included following careful examination.

### Inclusion Criteria

The included studies needed to have met the following criteria: (1) only the case–control studies or cohort studies were considered, (2) evaluated the XRCC1 Arg399Gln polymorphism and the risk of cancer, and (3) the genotype distribution of the polymorphisms in cases and controls were described in details and the results were expressed as odds ratio (OR) and corresponding 95% confidence interval (95% CI). Major reasons for exclusion of studies were as follows: (1) not for cancer research, (2) only case population, and (3) duplicate of previous publication.

### Data Extraction

Information was carefully extracted from all eligible studies independently by two investigators according to the inclusion criteria listed above. The following data were collected from each study: first author’s name, year of publication, country of origin, ethnicity, source of controls, sample size, and numbers of cases and controls in the XRCC1 Arg399Gln genotypes whenever possible. Ethnicity was categorized as “Caucasian,” “African,” (including African Americans) and “Asian.” We considered the samples of studies from India and Pakistan as of “Indian” ethnicity, and samples from Middle Eastern countries as “Middle Eastern” ethnicity. When one study did not state which ethnic groups was included or if it was impossible to separate participants according to phenotype, the sample was termed as “mixed population.” Meanwhile, studies investigating more than one kind of cancer were counted as individual data set only in subgroup analyses by cancer type. We did not define any minimum number of patients to include in this meta-analysis. Articles that reported different ethnic groups and different countries or locations, we considered them different study samples for each category cited above.

### Statistical Analysis

Crude odds ratios (ORs) together with their corresponding 95% CIs were used to assess the strength of association between the XRCC1 Arg399Gln polymorphism and the risk of cancer. Following published recommendations for quality assessment in meta-analyses of genetic associations, we examined: choice of genetic models (we adopted three genetic models, avoiding assuming only one “wrong” genetic model). The pooled ORs were performed for dominant model (Arg/Gln+Gln/Gln *versus* Arg/Arg); recessive model (Gln/Gln *versus* Arg/Gln+Arg/Arg); additive model (Gln/Gln *versus* Arg/Arg), respectively. Between-study heterogeneity was assessed by calculating *Q*-statistic (Heterogeneity was considered statistically significant if *P*<0.10) [13] and quantified using the *I* ^2^ value, a value that describes the percentage of variation across studies that are due to heterogeneity rather than chance, where *I* ^2^ = 0% indicates no observed heterogeneity, with 25% regarded as low, 50% as moderate, and 75% as high [14]. If results were not heterogeneous, the pooled ORs were calculated by the fixed-effect model (we used the *Q*-statistic, which represents the magnitude of heterogeneity between-studies) [15]. Otherwise, a random-effect model was used (when the heterogeneity between-studies were significant) [16]. In addition to the comparison among all subjects, we also performed stratification analyses by cancer type (if one cancer type contained less than three individual studies, it was combined into the “other cancers” group), source of control, and ethnicity. Moreover, the extent to which the combined risk estimate might be affected by individual studies was assessed by consecutively omitting every study from the meta-analysis (leave-one-out sensitivity analysis). This approach would also capture the effect of the oldest or first positive study (first study effect). In addition, we also ranked studies according to sample size, and then repeated this meta-analysis. Sample size was classified according to a minimum of 200 participants and those with fewer than 200 participants. The cite criteria were previously described [17]. Last, sensitivity analysis was also performed, excluding studies whose allele frequencies in controls exhibited significant deviation from the Hardy–Weinberg equilibrium (HWE), given that the deviation may denote bias. Deviation of HWE may reflect methodological problems such as genotyping errors, population stratification or selection bias. HWE was calculated by using the goodness-of-fit test, and deviation was considered when *P*<0.01. Begg’s funnel plots [18] and Egger’s linear regression test [19] were used to assess publication bias. A meta-regression analysis was carried out to identify the major sources of between-studies variation in the results, using the log of the ORs from each study as dependent variables, and cancer type, ethnicity, sample size, and source of controls as the possible sources of heterogeneity. All of the calculations were performed using STATA version 10.0 (STATA Corporation, College Station, TX).

## Results

### Eligible Studies and Meta-analysis Databases

**Figure 1:**
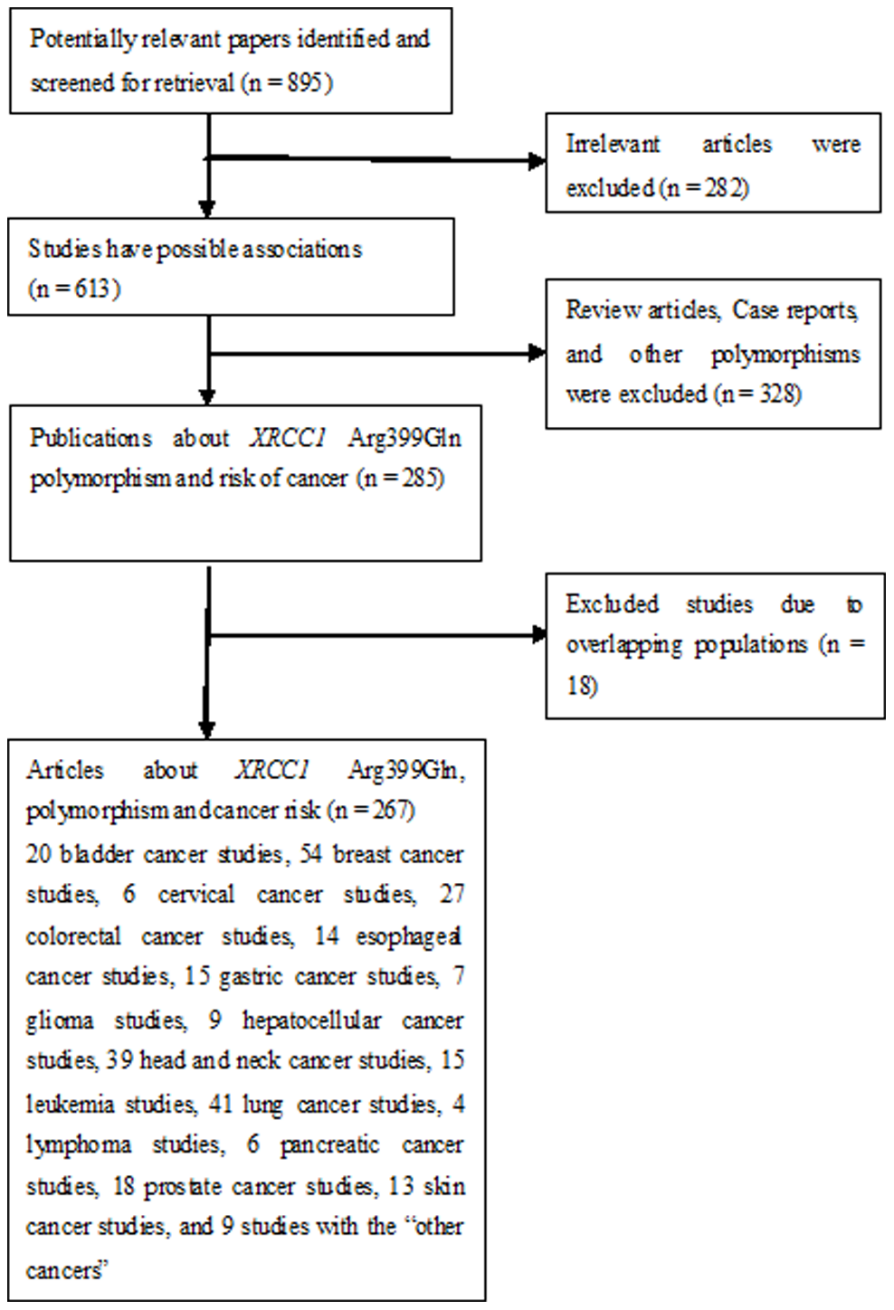
Study flow chart explaining the selection of the 297 eligible case–control studies included in the meta-analysis.

**Fig. 1** graphically illustrates the trial flow chart. A total of 895 articles regarding XRCC1 polymorphisms with respect to cancer were identified. After screening the titles and abstracts, 610 articles were excluded because they were review articles, case reports, other polymorphisms of XRCC1, or irrelevant to the current study. In addition, of these published articles, 18 publications (16, 23, 70, 90, 102, 106, 118, 144, 174, 190, 195, 196, 217, 224, 245, 256, 261, 263 in References S1) were excluded because of their populations overlapped with another 18 included studies (15, 17, 18, 45, 63, 101, 125, 131, 145, 149, 150, 156, 191, 200, 199, 203, 226, 242 in References S1). As summarized in **Table S1**, 267 publications with 297 case–control studies were selected among the meta-analysis, including 93,941 cases and 121,480 controls. Among these studies, one study was included in the recessive model and nine studies were included in the dominant model only because they provided the genotypes of Arg/Gln+Arg/Arg *versus* Gln/Gln and Arg/Gln+Gln/Gln *versus* Arg/Arg as a whole, respectively. In addition, there were 20 bladder cancer studies, 54 breast cancer studies, six cervical cancer studies, 27 colorectal cancer studies, 14 esophageal cancer studies, 15 gastric cancer studies, seven glioma studies, nine hepatocellular cancer studies, 39 head and neck cancer studies, 15 leukemia studies, 41 lung cancer studies, four lymphoma studies, six pancreatic cancer studies, 18 prostate cancer studies, 13 skin cancer studies, and nine studies with the “other cancers”. All of the cases were pathologically confirmed.

### Quantitative Synthesis

The evaluations of the association of XRCC1 Arg399Gln polymorphism with cancer risk are shown in **Table 1**. Overall, significantly increased cancer risk was observed in any genetic model (dominant model: OR = 1.04, 95% CI = 1.01–1.07, *P* value of heterogeneity test [*P*_h_] <0.001, *I* ^2^ = 52.6%; recessive model: OR = 1.08, 95% CI = 1.03–1.13, *P*_h_<0.001, *I* ^2^ = 48.8%; additive model: OR = 1.09, 95% CI = 1.04–1.14, *P*_h_<0.001, *I* ^2^ = 49.4%). However, there was significant heterogeneity between studies. Hence, we then performed the subgroup analysis by cancer type. We found that individuals with the minor variant genotypes had a higher risk of breast cancer (recessive model: OR = 1.09, 95% CI = 1.00–1.18, *P*_h_<0.001, *I* ^2^ = 50.6%; additive model: OR = 1.10, 95% CI = 1.01–1.20, *P*_h_<0.001, *I* ^2^ = 49.1%), cervical cancer (recessive model: OR = 1.37, 95% CI = 1.03–1.81, *P*_h_ = 0.765, *I* ^2^ = 0.0%; additive model: OR = 1.37, 95% CI = 1.02–1.84, *P*_h_ = 0.134, *I* ^2^ = 43.1%), colorectal cancer (recessive model: OR = 1.18, 95% CI = 1.00–1.39, *P*_h_ = 0.001, *I* ^2^ = 54.2%; additive model: OR = 1.18, 95% CI = 1.00–1.42, *P*_h_<0.001, *I* ^2^ = 57.4%), and leukemia (dominant model: OR = 1.24, 95% CI = 1.00–1.53, *P*_h_<0.001, *I* ^2^ = 66.8%), as shown in **Table 1**. Significantly decreased bladder cancer risk was found to be associated with the minor variant genotypes in recessive model (OR = 0.87, 95% CI = 0.78–0.97, *P*_h_ = 0.430, *I* ^2^ = 2.1%). For the breast cancer studies, we also performed the subgroup analysis by menopausal status, no significant association was observed in premenopausal and postmenopausal women (data not shown). We also performed the subgroup analysis by smoker habits for the lung cancer studies, no significant association was found among smokers and non-smokers (data not shown).

**Table 1:** Stratified analysis of XRCC1 Arg399Gln polymorphism on cancer risk^1^.

Variables	No. comparisons(SZ case/control)	Dominant model		Recessive model		Additive model	
		OR (95% CI)	*P*_h_ */I* 2	OR (95% CI)	*P*_h_ */I* 2	OR (95% CI)	*P*_h_ */I* 2
Overall	297 (93,941/121,480)	1.04 (1.01–1.07)*	<0.001/52.6%	1.08 (1.03–1.13)*	<0.001/48.8%	1.09 (1.04–1.14)*	<0.001/49.4%
Cancer type							
Bladder cancer	20 (6,376/7,393)	1.02 (0.95**–**1.09)	0.256/15.9%	**0.87 (0.78–0.97)**	0.430/2.1%	0.90 (0.80**–**1.01)	0.335/10.2%
Breast cancer	54 (29,549/32,619)	1.05 (0.99**–**1.10)*	<0.001/46.8%	1.09 (1.00–1.18)*	<0.001/50.6%	1.10 (1.01–1.20)*	<0.001/49.1%
Cervical cancer	6 (1,025/1,690)	1.00 (0.71**–**1.41)*	0.005/70.3%	**1.37 (1.03–1.81)**	0.765/0.0%	1.37 (1.02**–**1.84)	0.134/43.1%
Colorectal cancer	27 (7,919/12,385)	1.07 (0.96**–**1.18)*	0.001/53.6%	1.18 (1.00–1.39)*	0.001/54.2%	1.18 (1.00–1.42)*	<0.001/57.4%
Esophageal cancer	14 (3,166/6,244)	0.99 (0.88**–**1.12)*	0.050/41.9%	1.13 (0.93**–**1.37)*	0.061/40.0%	1.13 (0.90**–**1.41)*	0.019/49.4%
Gastric cancer	15 (3,382/7,282)	1.00 (0.86**–**1.15)*	0.002/59.1%	1.08 (0.94**–**1.25)	0.479/0.0%	1.07 (0.93**–**1.25)	0.155/27.3%
Glioma	7 (2,487/3,629)	2	<0.001/87.8%	2	<0.001/79.9%	2	<0.001/88.1%
Hepatocellularcancer	9 (1,621/2,310)	1.18 (0.92**–**1.50)*	0.009/60.5%	1.13 (0.90**–**1.42)	0.978/0.0%	1.23 (0.96**–**1.59)	0.829/0.0%
Head and neckcancer	39 (8,535/12,255)	0.99 (0.91**–**1.08)*	<0.001/52.2%	0.97 (0.86**–**1.09)*	0.035/31.3%	0.97 (0.84**–**1.11)*	0.004/41.5%
Leukemia	15 (2,261/2,854)	1.24 (1.00–1.53)*	<0.001/66.8%	1.09 (0.91**–**1.30)	0.206/22.8%	1.23 (0.91**–**1.67)*	0.009/53.5%
Lung cancer	41 (14,156/16,667)	1.00 (0.94–1.07)*	0.009/37.9%	1.05 (0.94–1.18)*	0.017/36.4%	1.05 (0.93–1.19)*	0.003/43.3%
Lymphoma	4 (827/1,414)	1.10 (0.92**–**1.32)	0.802/0.0%	1.15 (0.86**–**1.54)	0.759/0.0%	1.20 (0.88**–**1.64)	0.773/0.0%
Pancreatic cancer	6 (1,247/2,222)	0.99 (0.86**–**1.15)	0.293/18.5%	1.04 (0.83**–**1.29)	0.783/0.0%	1.03 (0.82**–**1.30)	0.510/0.0%
Prostate cancer	18 (4,452/4,431)	1.05 (0.89**–**1.23)*	<0.001/63.0%	1.20 (0.99**–**1.45)*	0.028/43.7%	1.18 (0.97**–**1.43)*	0.093/32.9%
Skin cancer	13 (4,763/5,471)	0.99 (0.91**–**1.07)	0.476/0.0%	0.95 (0.84**–**1.07)	0.170/27.2%	0.95 (0.84**–**1.08)	0.305/13.9%
Other cancer	9 (2,175/2,614)	2	<0.001/78.7%	2	<0.001/89.7%	2	<0.001/84.4%

1 all summary ORs were calculated using fixed-effects models. In the case of significant heterogeneity (indicated by *), ORs were calculated using random-effects models;

2 the results were excluded due to high heterogeneity; the bold values indicate that the results are statistically significant.

### Ethnicity and Cancer Risk Attributed to the XRCC1 Arg399Gln Polymorphism

We further examined the association of the XRCC1 Arg399Gln polymorphism and cancer risk according to cancer type and ethnicity (**Table 2**) because there was significant heterogeneity between studies. For the samples of Caucasians, no significant association was observed in any genetic model. For the samples of Asians, we found that individuals with the minor variant genotypes had a higher risk of breast cancer (recessive model: OR = 1.20, 95% CI = 1.04–1.39, *P*_h_ = 0.339, *I* ^2^ = 11.5%; additive model: OR = 1.18, 95% CI = 1.02–1.37, *P*_h_ = 0.269, *I* ^2^ = 19.5%), hepatocellular cancer (dominant model: OR = 1.39, 95% CI = 1.06–1.84, *P*_h_ = 0.040, *I* ^2^ = 60.0%), and prostate cancer (recessive model: OR = 1.43, 95% CI = 1.02–2.00, *P*_h_ = 0.383, *I* ^2^ = 1.9%; additive model: OR = 1.55, 95% CI = 1.02–2.33, *P*_h_ = 0.388, *I* ^2^ = 0.8%). For the samples of Africans, significant association was only observed among breast cancer (dominant model: OR = 1.28, 95% CI = 1.07–1.54, *P*_h_ = 0.348, *I* ^2^ = 9.1%; additive model: OR = 1.81, 95% CI = 1.08–3.02, *P*_h_ = 0.988, *I* ^2^ = 0.0%). For the samples of Indians, significant association was also observed among breast cancer (dominant model: OR = 1.39, 95% CI = 1.06–1.84, *P*_h_ = 0.040, *I* ^2^ = 60.0%; recessive model: OR = 1.43, 95% CI = 1.02–2.00, *P*_h_ = 0.383, *I* ^2^ = 1.9%; additive model: OR = 1.55, 95% CI = 1.02–2.33, *P*_h_ = 0.388, *I* ^2^ = 0.8%) and prostate cancer (dominant model: OR = 1.26, 95% CI = 1.00–1.58, *P*_h_ = 0.207, *I* ^2^ = 36.5%).

**Table 2:** Summary ORs (95% CI) categorized by ethnicity for the XRCC1 Arg399Gln polymorphism under different genetic models and cancer type^1^.

Ethnicity	Cancer type^3^	No. comparisons(SZ case/control)	Dominant model		Recessive model		Additive model	
			OR (95% CI)	*P*_h_ */I* 2	OR (95% CI)	*P*_h_ */I* 2	OR (95% CI)	*P*_h_ */I* 2
Asian	Bladder cancer	4 (956/1,388)	0.86 (0.73**–**1.02)	0.401/0.0%	**0.74 (0.56–0.99)**	0.412/0.0%	**0.71 (0.53–0.96)**	0.421/0.0%
	Breast cancer	9 (4,804/5,522)	1.00 (0.93**–**1.08)	0.225/24.6%	**1.20 (1.04–1.39)**	0.339/11.5%	**1.18 (1.02–1.37)**	0.269/19.5%
	Cervical cancer	3 (715/1,238)	1.07 (0.88**–**1.29)	0.715/0.0%	1.23 (0.83**–**1.82)	0.841/0.0%	1.26 (0.85**–**1.88)	0.822/0.0%
	Colorectal cancer	7 (2,662/4,541)	1.07 (0.89**–**1.30)*	0.003/69.4%	1.19 (0.84**–**1.70)*	0.009/67.2%	1.22 (0.82**–**1.82)*	0.002/72.8%
	Esophageal cancer	6 (1,721/2,726)	1.04 (0.82**–**1.32)*	0.006/69.6%	1.33 (0.91**–**1.94)*	0.023/61.7%	1.37 (0.88**–**2.14)*	0.006/69.5%
	Gastric cancer	5 (1,667/2,322)	2	<0.001/83.9%	1.06 (0.83**–**1.32)	0.545/0.0%	1.01 (0.69**–**1.48)*	0.070/53.9%
	Hepatocellularcancer	5 (1,260/1,557)	1.39 (1.06–1.84)*	0.040/60.0%	1.14 (0.88**–**1.46)	0.881/0.0%	1.32 (0.99**–**1.76)	0.733/0.0%
	Head and neckcancer	9 (1,718/2,018)	1.02 (0.81**–**.28)*	0.009/61.0%	1.13 (0.88**–**1.45)	0.233/23.7%	1.11 (0.75**–**1.64)*	0.058/46.9%
	Leukemia	3 (431/883)	2	0.001/85.6%	0.94 (0.55**–**1.60)	0.161/49.2%	1.20 (0.37**–**3.89)*	0.037/76.9%
	Lung cancer	17 (6,010/6,550)	1.07 (0.97**–**1.19)*	0.031/42.9%	1.14 (0.94**–**1.39)*	0.053/39.4%	1.16 (0.93**–**1.46)*	0.013/49.5%
	Prostate cancer	4 (669/762)	1.17 (0.93**–**1.46)	0.110/50.3%	**1.43 (1.02–2.00)**	0.383/1.9%	**1.55 (1.02–2.33)**	0.388/0.8%
Caucasian	Bladder cancer	13 (4,834/5,198)	1.02 (0.94**–**1.11)	0.685/0.0%	0.91 (0.81**–**1.04)	0.736/0.0%	0.94 (0.82**–**1.07)	0.650/0.0%
	Breast cancer	27 (18,056/18,909)	0.99 (0.94**–**1.05)*	0.036/35.6%	0.98 (0.92**–**1.04)	0.315/10.1%	0.99 (0.92**–**1.05)	0.182/19.6%
	Colorectal cancer	14 (2,191/4,413)	1.02 (0.87**–**1.20)*	0.067/39.1%	1.18 (0.91**–**1.54)*	0.023/48.1%	1.15 (0.86**–**1.53)*	0.018/49.6%
	Esophageal cancer	4 (568/1,233)	0.87 (0.71**–**1.07)	0.786/0.0%	1.01 (0.74**–**1.38)	0.675/0.0%	0.95 (0.68**–**1.32)	0.786/0.0%
	Gastric cancer	8 (1,252/3,473)	0.97 (0.85**–**1.12)	0.317/14.4%	1.05 (0.85**–**1.30)	0.408/2.8%	1.04 (0.83**–**1.30)	0.385/5.9%
	Glioma	6 (2,216/3,340)	2	<0.001/90.0%	2	<0.001/81.2%	2	<0.001/90.0%
	Head and neckcancer	20 (4,785/7,185)	1.02 (0.94**–**1.10)	0.164/23.7%	0.93 (0.83**–**1.05)	0.223/18.6%	0.95 (0.84**–**1.08)	0.237/17.4%
	Leukemia	10 (1,685/1,716)	1.14 (0.92**–**1.41)*	0.023/53.3%	1.07 (0.87**–**1.30)	0.182/28.5%	1.17 (0.83**–**1.67)*	0.013/57.1%
	Lung cancer	19 (7,308/9,140)	0.98 (0.92–1.04)	0.560/0.0%	1.00 (0.87–1.16)*	0.054/38.4%	0.99 (0.89–1.10)	0.120/29.7%
	Prostate cancer	7 (2,790/2,507)	1.05 (0.86**–**1.27)*	0.042/54.1%	1.00 (0.84**–**1.18)	0.452/0.0%	0.96 (0.80**–**1.15)	0.216/29.1%
	Skin cancer	8 (3,361/3,548)	0.94 (0.85**–**1.04)	0.907/0.0%	0.93 (0.81**–**1.08)	0.212/27.1%	0.91 (0.78**–**1.06)	0.442/0.0%
African	Breast cancer	4 (1,166/1,116)	**1.28 (1.07–1.54)**	0.348/9.1%	1.59 (0.96**–**2.64)	0.918/0.0%	**1.81 (1.08–3.02)**	0.988/0.0%
	Lung cancer	3 (524/644)	1.04 (0.81**–**1.35)	0.682/0.0%	0.79 (0.39**–**1.62)	0.603/0.0%	0.80 (0.39**–**1.64)	0.645/0.0%
Indian	Breast cancer	3 (632/715)	**1.64 (1.31–2.04)**	0.461/0.0%	1.94 (1.09–3.47)*	0.037/69.8%	2.06 (1.50**–**2.84)	0.230/31.9%
	Head and neckcancer	3 (697/773)	2	<0.001/89.9%	2	0.012/77.3%	2	<0.001/86.0%
	Prostate cancer	3 (516/750)	**1.26 (1.00–1.58)**	0.207/36.5%	2	0.001/86.6%	1.30 (0.73**–**2.31)*	0.024/73.0%
Mixed	Breast cancer	11 (4,891/6,357)	1.10 (0.98**–**1.23)*	0.074/41.2%	1.00 (0.78**–**1.29)*	<0.001/73.5%	1.07 (0.83**–**1.36)*	<0.001/68.6%
	Colorectal cancer	4 (2,716/3,092)	0.99 (0.89**–**1.10)	0.682/0.0%	1.17 (0.79**–**1.73)*	0.017/70.7%	1.14 (0.79**–**1.65)*	0.040/63.8%
	Esophageal cancer	3 (757/2,125)	1.06 (0.89**–**1.26)	0.417/0.0%	1.17 (0.91**–**1.51)	0.805/0.0%	1.18 (0.90–1.55)	0.662/0.0%
	Head and neckcancer	3 (592/1,430)	0.81 (0.67**–**0.99)	0.334/8.8%	2	0.014/76.7%	0.80 (0.43**–**1.50)*	0.024/73.1%
	Skin cancer	3 (996/1,625)	1.06 (0.89**–**1.25)	0.383/0.0%	1.08 (0.84**–**1.39)	0.470/0.0%	1.11 (0.85**–**1.46)	0.485/0.0%

1 all summary ORs were calculated using fixed-effects models. In the case of significant heterogeneity (indicated by *), ORs were calculated using random-effects models;

2 the results were excluded due to high heterogeneity;

3 total bladder cancer comparisons add up to 17 (which should be 20) because 1 Africans study, 1 Indians study, and 1 mixed population study did not be included. The reason is same with breast cancer, colorectal cancer, hepatocellular cancer and so on; the bold values indicate that the results are statistically significant; SZ, sample size

### Source of Controls and Cancer Risk Attributed to the XRCC1 Arg399Gln Polymorphism

We also examined the association of the XRCC1 Arg399Gln polymorphism and cancer risk according to cancer type and source of controls (**Table 3**). For the population-based studies, the XRCC1 Arg399Gln polymorphism was associated with breast cancer and bladder cancer risk. For the hospital-based studies, significant association was observed among bladder cancer, breast cancer, cervical cancer, colorectal cancer, leukemia, and prostate cancer.

**Table 3:** Summary ORs (95% CI) and value of value of the heterogeneity of XRCC1 Arg399Gln polymorphism for studies according to source of controls and cancer type^1^.

Source of controls		Cancer type^3^	No. comparisons(SZ case/control)		Dominant model			Recessive model			Additive model		
					OR (95% CI)		*P*_h_ */I* 2	OR (95% CI)	*P*_h_ */I* 2		OR (95% CI)	*P*_h_ */I* 2	
Population-based studies		Bladder cancer	3 (628/949)		**1.32 (1.07–1.63)**		0.674/0.0%	0.80 (0.59**–**1.08)*	0.095/57.6%		0.97 (0.70**–**1.34)	0.113/54.1%	
		Breast cancer	29 (22,399/24,221)		1.04 (0.99**–**1.08)		0.136/22.8%	1.06 (0.97**–**1.17)*	<0.001/55.4%		1.07 (0.97**–**1.17)*	0.003/47.2%	
		Colorectal cancer	8 (3,700/7,176)		1.02 (0.89**–**1.16)*		0.089/43.4%	1.20 (0.85**–**1.71)*	<0.001/77.7%		1.20 (0.84**–**1.71)*	<0.001/77.7%	
		Esophageal cancer	8 (2,256/4,785)		0.98 (0.85**–**1.13)*		0.080/44.8%	1.16 (0.97**–**1.38)	0.497/0.0%		1.15 (0.95**–**1.38)	0.252/22.2%	
		Gastric cancer	9 (2,352/5,836)		0.98 (0.80**–**1.20)*		<0.001/73.6%	1.09 (0.92**–**1.30)	0.319/13.8%		1.06 (0.83**–**1.34)*	0.065/45.6%	
		Glioma	3 (1,438/2,340)		1.02 (0.87**–**1.18)		0.627/0.0%	1.02 (0.75**–**1.39)*	0.064/63.7%		1.18 (0.92**–**1.50)	0.976/0.0%	
		Head and neck cancer	5 (990/2,523)		1.05 (0.80**–**1.37)		0.862/0.0%	1.01 (0.78**–**1.30)	0.923/0.0%		1.04 (0.88**–**1.22)	0.701/0.0%	
		Lung cancer	18 (5,943/7,925)		0.94 (0.87**–**1.01)		0.116/29.5%	1.01 (0.90**–**1.14)	0.145/27.1%		0.97 (0.86**–**1.10)	0.119/29.8%	
		Pancreatic cancer	3 (293/919)		1.15 (0.88**–**1.50)		0.665/0.0%	1.23 (0.82**–**1.85)	0.995/0.0%		1.29 (0.84**–**1.98)	0.820/0.0%	
		Prostate cancer	6 (2,017/2,192)		0.97 (0.80**–**1.18)		0.138/40.1%	1.23 (0.81**–**1.86)*	0.050/57.9%		1.00 (0.81**–**1.22)	0.102/48.3%	
		Skin cancer	3 (1,456/1,683)		1.01 (0.86**–**1.18)		0.299/17.2%	0.84 (0.68**–**1.04)	0.156/46.2%		0.86 (0.68**–**1.09)	0.132/50.6%	
Hospital-based studies		Bladder cancer	17 (5,748/6,444)		0.99 (0.92**–**1.06)		0.512/0.0%	**0.88** **(0.79–0.99)**	0.585/0.0%		0.89 (0.79**–**1.01)	0.430/1.8%	
		Breast cancer	24 (6,625/7,774)		1.11 (0.99**–**1.25)*		<0.001/62.5%	**1.16** (1.00–1.35)*	0.014/43.2%		**1.22** (1.02–1.45)*	0.002/50.7%	
		Cervical cancer	5 (894/1370)		0.94 (0.61**–**1.44)*		0.003/75.3%	**1.39** **(1.02–1.88)**	0.624/0.0%		1.27 (0.73**–**2.18)*	0.071/57.3%	
		Colorectal cancer	18 (3,914/4,849)		1.11 (0.95**–**1.29)*		0.001/59.2%	**1.23** **(1.03–1.42)**	0.249/17.5%		**1.24** (1.06–1.45)*	0.110/30.9%	
		Esophageal cancer	6 (910/1,459)		1.00 (0.78**–**1.28)*		0.095/46.7%	1.09 (0.67**–**1.76)*	0.010/66.9%		1.08 (0.63**–**1.85)*	0.005/70.0%	
		Gastric cancer	6 (1,030/1,446)		1.01 (0.85**–**1.19)		0.578/0.0%	1.06 (0.77**–**1.46)	0.508/0.0%		1.09 (0.78**–**1.51)	0.471/0.0%	
		Glioma	4 (1,049/1,289)		2		<0.001/92.3%	2	<0.001/86.9%		2	<0.001/92.8%	
		Hepatocellular cancer	9 (1,621/2,310)		1.18 (0.92**–**1.50)*		0.009/60.5%	1.13 (0.90**–**1.42)	0.978/0.0%		1.23 (0.96**–**1.59)	0.829/0.0%	
		Head and neck cancer	34 (7,545/9,732)		0.99 (0.89**–**1.09)*		<0.001/57.0%	0.96 (0.83**–**1.10)*	0.011/39.2%		0.96 (0.82**–**1.13)*	0.001/48.0%	
		Leukemia	15 (2,261/2,854)		**1.24** (1.00–1.53)*		<0.001/66.8%	1.09 (0.91**–**1.30)	0.206/22.8%		1.23 (0.91**–**1.67)*	0.009/53.5%	
		Lung cancer	23 (8,213/8,742)		1.07 (0.98**–**1.16)*		0.062/33.4%	1.15 (0.98**–**1.34)*	0.015/44.7%		1.17 (0.98**–**1.40)*	0.004/50.9%	
		Lymphoma	3 (399/821)		1.06 (0.83**–**1.37)		0.655/0.0%	1.35 (0.84**–**2.18)	0.797/0.0%		1.34 (0.82**–**2.19)	0.669/0.0%	
HB	Pancreatic cancer		3 (954/1,303)	0.93 (0.78**–**1.11)		0.158/45.7%		0.97 (0.75**–**1.25)	0.671/0.0%	0.94 (0.71**–**1.24)		0.407/0.0%	
	Prostate cancer		10 (1,798/1,759)	1.06 (0.81**–**1.39)*		0.001/68.8%		1.24 (0.94**–**1.63)*	0.043/48.3%	**1.28** **(1.04–1.58)**		0.184/28.3%	
	Skin cancer		10 (3,307/3,788)	0.98 (0.89**–**1.08)		0.424/1.5%		1.01 (0.87**–**1.17)	0.294/16.2%	0.99 (0.85**–**1.16)		0.450/0.0%	

1 all summary ORs were calculated using fixed-effects models. In the case of significant heterogeneity (indicated by *), ORs were calculated using random-effects models;

2 the results were excluded due to high heterogeneity;

3 total breast cancer comparisons add up to 53 (which should be 54) because there was one study that we can not determine hospital-based study, population-based study or family-based study. The reason is same with colorectal cancer, prostate cancer and so on; PB, population-based study; HB, hospital-based study; SZ Sample size. The bold values indicate that the results are statistically significant.

### Anatomical Site, Histological Type, and Association of the XRCC1 Arg399Gln Polymorphism with Cancer Risk

We next completed a subgroup analysis by tumor site and histological type or anatomical location (**Table 4**). Overall, there was no association between the XRCC1 Arg399Gln polymorphism and risk of nasopharyngeal cancer, oral cancer, larynx cancer, thyroid cancer, and other head and neck cancer sites. For the lung and gastric cancers, no significant association was observed among lung adenocarcinoma, lung squamous cell carcinoma, small cell lung cancer, and cardia gastric cancer.

**Table 4:** Summary ORs (95% CI) for the XRCC1 Arg399Gln polymorphism categorized by histological type or anatomical area in a specific tumor site^1^.

Cancer type	Histological type oranatomical area	No. comparisons(SZ case/control)	Dominant model		Recessive model		Additive model	
			OR (95% CI)	*P*_h_/*I* 2	OR (95% CI)	*P*_h_/*I* 2	OR (95% CI)	*P*_h_/*I* 2
		4 (1,424/1,428)	1.03 (0.89**–**1.19)	0.278/22.1%	1.25 (0.94**–**1.67)	0.992/0.0%	1.26 (0.94**–**1.69)	0.974/0.0%
	Oral	3 (525/659)	2	<0.001/87.1%	2	0.004/82.3%	2	0.001/86.8%
	Larynx	4 (706/822)	1.39 (0.95**–**2.02)*	0.036/64.9%	1.17 (0.74**–**1.84)	0.100/52.0%	1.38 (0.81**–**2.34)*	0.067/58.1%
	Thyroid	9 (1,705/2,882)	0.87 (0.74**–**1.03)*	0.092/41.2%	0.89 (0.73**–**1.09)	0.362/8.8%	0.84 (0.68**–**1.04)	0.253/21.4%
	Other sites^3^	17 (3,916/5,868)	0.98 (0.87**–**1.10)*	0.041/40.8%	0.90 (0.76**–**1.08)*	0.099/32.1%	0.91 (0.75**–**1.10)*	0.081/34.5%
Lung cancer	AC^4^	11 (1,821/5,536)	1.13 (0.92**–**1.39)*	0.002/64.4%	1.31 (0.92**–**1.87*)	0.001/66.7%	1.34 (0.89**–**2.03)*	<0.001/73.3%
	SC^5^	6 (1,688/4,014)	0.97 (0.75**–**1.26)*	0.006/69.4%	1.06 (0.89**–**1.27)	0.225/29.5%	1.10 (0.77**–**1.57)*	0.058/56.1%
	SCLC^6^	3 (112/879)	0.75 (0.37**–**1.55)*	0.088/58.8%	0.67 (0.32**–**1.43)	0.642/0.0%	0.62 (0.28**–**1.37)	0.997/0.0%
Gastric cancer	Cardia	6 (1,378/3,879)	2	<0.001/78.3%	1.25 (0.78**–**2.00)*	0.002/73.0%	1.21 (0.86**–**1.71)	0.100/45.8%

1 all summary ORs were calculated using fixed-effects models. In the case of significant heterogeneity (indicated by *), ORs were calculated using random-effects models;

2 the results were excluded due to high heterogeneity;

3 includes a diversity of head and neck cancer not separated by anatomical area in the studies analyzed;

4 means adenocarcinoma’.

5 means squamous cell carcinoma;

6 small cell lung cancer; the bold values indicate that the results are statistically significant.

### Test of Heterogeneity and Sensitivity

There was significant heterogeneity among these studies for dominant model comparison (*P*_h_<0.001), recessive model comparison (*P*_h_<0.001), and additive model comparison (*P*_h_<0.001). Then, we assessed the source of heterogeneity by ethnicity, cancer type, source of controls, and sample size. The results of meta-regression indicated that source of controls (dominant model: P = 0.241; recessive model: *P* = 0.626; additive model: *P* = 0.504), ethnicity (dominant model: P = 0.739; recessive model: *P* = 0.305; additive model: *P* = 0.334), cancer type (dominant model: *P* = 0.526; recessive model: *P* = 0.507; additive model: *P* = 0.848), and sample size (dominant model: *P* = 0.366; recessive model: *P* = 0.944; additive model: *P* = 0.665) did not contribute to substantial heterogeneity among the meta-analysis. Examining genotype frequencies in the controls, significant deviation from HWE was detected in the eight studies (7, 24, 69, 86, 93, 100, 169, 172 in References S1). When these studies were excluded, the result of XRCC1 Arg399Gln was changed among prostate cancer (recessive model: OR = 1.18, 95% CI = 1.04–1.35, *P*_h_ = 0.209, *I* ^2^ = 21.5%), as shown in **Table 5**. In addition, when this meta-analysis was performed excluding studies with small sample sizes, the results of XRCC1 Arg399Gln were changed among colorectal cancer (recessive model: OR = 1.18, 95% CI = 0.98–1.42, *P*_h_<0.001, *I* ^2^ = 62.9%; additive model: OR = 1.17, 95% CI = 0.97–1.43, *P*_h_<0.001, *I* ^2^ = 63.7%), hepatocellular cancer (dominant model: OR = 1.35, 95% CI = 1.05–1.75, *P*_h_ = 0.035, *I* ^2^ = 58.4%; additive model: OR = 1.39, 95% CI = 1.03–1.86, *P*_h_ = 0.954, *I* ^2^ = 0.0%), and leukemia (dominant model: OR = 1.18, 95% CI = 0.97–1.42, *P*_h_ = 0.012, *I* ^2^ = 55.8%), as shown in **Table 6**. In addition, after the study of Kelsey et al. (230 in References S1) was excluded, the results were changed among bladder cancer (recessive model: OR = 0.90, 95% CI = 0.80–1.01, *P*_h_ = 0.605, *I* ^2^ = 0.0%). After the study of Roszak et al. (22 in References S1) was excluded, the results were also changed among cervical cancer (recessive model: OR = 1.21, 95% CI = 0.86–1.70, *P*_h_ = 0.942, *I* ^2^ = 0.0%; additive model: OR = 1.11, 95% CI = 0.78–1.58, *P*_h_ = 0.517, *I* ^2^ = 0.0%). For the samples of Asians, when one study was excluded, the results were changed among bladder, breast and prostate cancers. For the samples of Africans, when one study was excluded, the results were also changed among breast cancer. For the samples of Indians, when one study was excluded, the results were also changed among prostate cancer. For the hospital-based studies, when one study was excluded, the results were changed among bladder cancer, cervical cancer, colorectal cancer, and leukemia. For the population-based studies, when one study was excluded, the results were also changed among bladder cancer.

**Table 5:** Summary ORs (95% CI) and value of the heterogeneity of XRCC1 Arg399Gln polymorphism under different genetic models according to studies with HWE on cancer risk^1^.

Variables^2^	No. comparisons(SZ case/control)	Dominant model		Recessive model		Additive model	
		OR (95% CI)	P_h_/I^2^	OR (95% CI)	P_h_/I^2^	OR (95% CI)	P_h_/I^2^
Overall	289 (92,485/119,277)	1.03 (1.00–1.06)*	<0.001/52.2%	1.09 (1.04–1.14)*	<0.001/47.8%	1.09 (1.04–1.15)*	<0.001/49.3%
Cancer type							
Breast cancer	52 (29,000/32,072)	1.03 (0.99**–**1.08)*	0.001/42.5%	**1.09 (1.01–1.18)**	<0.001/47.0%	1.10 (1.01–1.20)*	<0.001/47.4%
Bladder cancer	19 (6,216/6,811)	1.02 (0.95**–**1.09)	0.207/20.3%	**0.87 (0.78–0.97)**	0.360/8.2%	0.90 (0.80**–**1.02)	0.272/15.8%
Skin cancer	12 (4,566/5,378)	0.99 (0.91**–**1.08)	0.396/5.1%	0.97 (0.86**–**1.10)	0.331/11.7%	0.97 (0.85**–**1.11)	0.425/2.0%
Head and neckcancer	38 (8,424/12,155)	0.99 (0.91**–**1.09)*	<0.001/52.6%	0.97 (0.86**–**1.10)	0.030/32.5%	0.97 (0.85**–**1.12)	0.004/42.5%
Prostate cancer	17 (4,281/4,231)	1.03 (0.87**–**1.21)*	<0.001/63.0%	**1.18 (1.04–1.35)**	0.209/21.5%	1.13 (0.98**–**1.31)	0.119/30.5%
Hepatocellular cancer	8 (1,558/2,021)	1.23 (0.96**–**1.58)*	0.017/59.0%	1.13 (0.90**–**1.43)	0.952/0.0%	1.25 (0.96**–**1.63)	0.761/0.0%
Esophageal cancer	12 (2,856/5,588)	0.98 (0.90**–**1.08)	0.317/13.0%	1.14 (0.92**–**1.42)	0.033/47.7%	1.12 (0.88**–**1.42)	0.019/51.6%

1 all summary ORs were calculated using fixed-effects models. In the case of significant heterogeneity (indicated by *), ORs were calculated using random-effects models;

2 examine genotype frequencies in the controls, significant deviation from HWE was detected in the eight studies. These studies were only breast cancer, bladder cancer, skin cancer, head and neck cancer, prostate cancer, hepatocellular cancer, esophageal cancer. Hence, only these cancers were analyzed. Cancer comparisons add up to 158 (which should be 289) because the remaining 131 comparisons were 6 cervical cancer, 27 colorectal cancer, 15 gastric cancer, 7 glioma, 15 leukemia, 41 lung cancer, 4 lymphoma, 6 pancreatic cancer, and 9 other cancer; HWE hardy–weinberg equilibrium; the bold values indicate that the results are statistically significant.

**Table 6:** Summary ORs (95% CI) and value of the heterogeneity of XRCC1 Arg399Gln polymorphism under different genetic models according to studies with a minimum of 200 participants on cancer risk^1^.

Variables	No. comparisons(SZ case/control)	Dominant model		Recessive model		Additive model	
		OR (95% CI)	*P*_h_ */I* ^2^	OR (95% CI)	*P*_h_ */I* ^2^	OR (95% CI)	*P*_h_ */I* ^2^
Overall	263 (91,969/119,090)	1.03 (1.00–1.06)*	<0.001/51.0%	1.08 (1.03**–**1.13)*	<0.001/51.9%	1.09 (1.04–1.14)*	<0.001/50.9%
Cancer type							
Bladder cancer	17 (6,206/7,235)	1.02 (0.95**–**1.10)	0.234/18.8%	**0.88 (0.79–0.98)**	0.396/5.1%	0.91 (0.81**–**1.03)	0.306/13.2%
Breast cancer	49 (29,249/32,281)	1.04 (0.99**–**1.09)*	0.001/43.2%	1.08 (1.00–1.17)*	<0.001/52.2%	1.04 (1.00–1.18)*	<0.001/47.8%
Cervical cancer	5 (1,007/1,660)	^2^	0.003/75.6%	**1.37 (1.03–1.81)**	0.765/0.0%	**1.37 (1.02–1.84)**	0.134/43.1%
Colorectal cancer	20 (7,487/11,838)	1.04 (0.96**–**1.14)*	0.030/40.9%	1.18 (0.98**–**1.42)*	<0.001/62.9%	1.17 (0.97**–**1.43)*	<0.001/63.7%
Esophageal cancer	13 (3,110/6,149)	1.00 (0.88**–**1.13)*	0.040/45.0%	1.16 (0.95**–**1.41)*	0.065/40.4%	1.16 (0.92**–**1.45)*	0.021/49.9%
Gastric cancer	15 (3,382/7,282)	1.00 (0.86**–**1.15)*	0.002/59.1%	1.08 (0.94**–**1.25)	0.479/0.0%	1.07 (0.93**–**1.25)	0.155/27.3%
Hepatocellular cancer	6 (1,398/2,015)	1.35 (1.05–1.75)*	0.035/58.4%	1.19 (0.92**–**1.54)	0.952/0.0%	**1.39 (1.03–1.86)**	0.954/0.0%
Head and neck cancer	36 (8,308/12,035)	0.99 (0.91**–**1.07)*	0.002/45.3%	0.95 (0.84**–**1.08)*	0.025/34.3%	0.95 (0.83**–**1.10)*	0.006/41.3%
Leukemia	11 (2,027/2,525)	1.18 (0.97**–**1.42)*	0.012/55.8%	1.07 (0.89**–**1.29)	0.236/21.7%	1.17 (0.88**–**1.55)*	0.058/43.8%
Lymphoma	3 (794/1,362)	1.12 (0.93**–**1.34)	0.887/0.0%	1.16 (0.86**–**1.57)	0.575/0.0%	1.23 (0.90**–**1.69)	0.672/0.0%
Pancreatic cancer	4 (1,204/2,135)	0.99 (0.86**–**1.15)	0.145/44.4%	1.03 (0.83**–**1.29)	0.635/0.0%	1.03 (0.81**–**1.30)	0.349/8.8%
Prostate cancer	17 (4,387/4,388)	1.03 (0.88**–**1.21)*	<0.001/62.6%	1.20 (0.98**–**1.46)*	0.019/47.1%	1.18 (0.97**–**1.43)*	0.069/36.9%
Skin cancer	11 (4,680/5,354)	0.99 (0.91**–**1.07)	0.489/0.0%	0.95 (0.84**–**1.07)	0.127/33.9%	0.94 (0.83**–**1.07)	0.218/23.7%
Lung cancer	29 (10,512/12,692)	0.96 (0.89**–**1.03)*	0.078/28.5%	1.01 (0.93**–**1.10)	0.105/26.3%	0.99 (0.87**–**1.12)*	0.071/30.2%

1 All summary ORs were calculated using fixed-effects models. In the case of significant heterogeneity (indicated by *), ORs were calculated using random-effects models.

The bold values indicate that the results are statistically significant.

### Publication Bias

**Figure 2:**
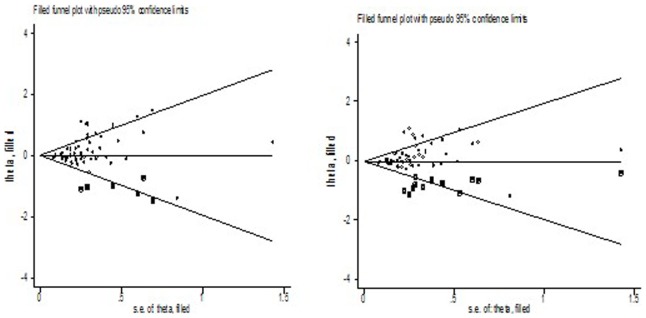
The Duval and Tweedie nonparametric “trim and fill” method’s funnel plot on breast cancer risk (recessive and additive model).

We performed Begg’s funnel plot and Egger’s test to assess the publication bias of literatures. Begg’s funnel plots and Egger’s test suggested that there might be publication bias in recessive model (*P* = 0.032) and additive model (*P* = 0.015) in overall cancer. Then, we examined if there was evidence of publication bias for studies in each cancer type group (**Table 1**). There were no asymmetries in the funnel plots (data not shown) and no statistical significance for Egger’s tests for most cancer sites, with the exception of breast cancer (recessive model: *P* = 0.008; additive model: *P* = 0.005). Their respective funnel plots indicated that the asymmetry was due mainly to a few studies with smaller sample sizes and large effect sizes, a fact more evident in the breast cancer group. Adjusting for possible publication bias using the Duval and Tweedie nonparametric “trim and fill” method for breast cancer, the results did not been changed between Arg399Gln polymorphism with the risk of breast cancer. **Figure 2** listed the Duval and Tweedie nonparametric “trim and fill” methods funnel plot in recessive model and additive model.

## Discussion

Cancer is the result of a series of DNA alternations in single cell or clone of that cell, which lead to loss of normal function, aberrant or uncontrolled cell growth and often metastases. BER is initiated by recognition and excision of damaged base by the specific DNA glycosylase. X-ray repair cross-complementing groups 1 (protein is a scaffold protein directly associated with polymerase beta, DNA ligase III, and poly (ADP-ribose) polymerase in a complex to facilitate the base excision repair (BER) and single-strand break repair (SSBR) processes [6], [20], [21]. A recent report provided data showing that the E2F1 transcription factor regulates XRCC1 and promotes DNA repair [22]. A XRCC1 deletion mutation in null homozygous mice is embryonic lethal [23]. XRCC1 has two BRCA1 carboxyl-terminal (BRCT) domains (BRCT1 and BRCT2), located centrally and at the C-terminal end, respectively. BRCT2 is responsible for binding and stabilizing DNA ligase III and is required for single-strand breaks and gaps repair (SSBR), specifically during the G0/G1 phases of the cell cycle [24]. The centre of BRCT1 domain binds to and down-regulates the single-strand breaks and gaps recognition protein PARP1 and is required for efficient SSBR during both G1 and S/G2 phases of the cell cycle. The polymorphism Arg399Gln is located close to BRCT1’s C-terminal boundary. The mutation in this domain will change XRCC1’s structure and may be disrupt the combination of BRCT1 and PARP1. Many studies have reported the association of XRCC1 Arg399Gln polymorphism with risk of cancer, however, the results remained controversial, although some original studies thought that Arg399Gln polymorphism was associated with risk of cancer, others had different opinions. In order to resolve this conflict, the meta-analysis of 297 eligible studies including 93,941 cases and 121,480 controls was performed to derive a more precise estimation of the association between XRCC1 Arg399Gln polymorphism and risk of different types of cancer.

Overall, our results show that XRCC1 Arg399Gln polymorphism is associated with increased cancer risk when all eligible studies were pooled into the meta-analysis. In further stratified and sensitivity analyses, significantly elevated hepatocellular and breast cancer risk was observed in Asians (dominant model: OR = 1.39, 95% CI = 1.06–1.84) and in Indians (dominant model: OR = 1.64, 95% CI = 1.31–2.04; recessive model: OR = 1.94, 95% CI = 1.09–3.47; additive model: OR = 2.06, 95% CI = 1.50–2.84), respectively. It should be considered that the apparent inconsistency of these results may underlie differences in ethnicity, lifestyle and disease prevalence as well as possible limitations due to the relatively small sample size. The current knowledge of carcinogenesis indicates a multi-factorial and multistep process that involves various genetic alterations and several biological pathways. Thus, it is unlikely that risk factors of cancer work in isolation from each other. And the same polymorphisms may play different roles in cancer susceptibility, because cancer is a complicated multi-genetic disease, and different genetic backgrounds may contribute to the discrepancy. And even more importantly, the low penetrance genetic effects of single polymorphism may largely depend on interaction with other polymorphisms and/or a particular environmental exposure. We observed a wide variation of the Gln allele frequencies of control resources in Asians (0.27), Indians (0.35), Caucasians (0.35) and Africans (0.17), and this different allele frequency might account for the association between the XRCC1 Arg399Gln polymorphism and cancer susceptibility among different ethnicity.

Based on biochemical properties described for XRCC1 polymorphism, we would expect that the Gln allele would be associated with higher susceptibility for all types of cancer. However, our results showed that such association was observed just for breast and hepatocellular cancer, suggesting that other factors may be modulating the XRCC1 polymorphism functionality. However, the exact mechanism for association between different tumor sites and XRCC1 Arg399Gln polymorphism was not clear, carcinogenetic mechanism may differ by different tumor sites and the XRCC1 genetic variants may exert varying effects in different cancers. Several previous meta-analyses assessed the association of XRCC1 Arg399Gln polymorphism with risk of breast, lung and hepatocellular cancer, and so on. Huang et al. [25] suggested that the Arg399Gln polymorphism were associated with an increased risk of breast cancer among Asians and Africans and while only slightly increased breast cancer risk in Caucasians. Saadat et al. [26] suggested that Arg399Gln polymorphism was associated with increased breast cancer risk in Asians. Li et al. [27] suggested that *XRCC1* Arg399Gln polymorphism may modify breast cancer risk in Caucasians and Asians. Wu et al. [11] concluded that *XRCC1* Arg399Gln is a risk factor for the development breast cancer, especially among Asians and Africans. Kiyohara et al. [28] suggested that the *XRCC1* Arg399Gln polymorphism was associated with an increased risk of lung cancer among Asians but not among Caucasians. Wang et al. [29] found a protective effect of the XRCC1 399 Gln/Gln and Arg/Gln or Gln/Gln polymorphisms for lung cancer on the basis of population control (OR = 0.73, 95% CI: 0.58–0.92; OR = 0.86, 95% CI: 0.77–0.97, respectively). Dai et al. [9] found that *XRCC1* Arg399Gln polymorphism may be association with risk of lung cancer. Liu et al. [30], Zhang et al. [31], and Xie et al. [32] suggested that the XRCC1 Arg399Gln polymorphism was not associated with the risk of hepatocellular cancer. Li et al. [33] indicated that the Arg399Gln polymorphisms of XRCC1 may be a genetic susceptibility for HCC in the East Asians. Duan et al. [34] indicated that the XRCC1 Arg399Gln gene polymorphism is associated with an increased hepatocellular carcinoma risk In the Chinese Han populations. Our meta-analysis should be more stringent and comprehensive. Firstly, more up to date studies were recruited to provide statistically significant results. Secondly, the association of Arg399Gln, with risk of cancer had been explored in detail. Present meta-analysis suggests the participation of XRCC1 Arg399Gln is a genetic susceptibility for hepatocellular cancer in Asians, breast cancer in Indians, and is not associated with lung cancer risk.

In the present meta-analysis, highly between-studies heterogeneity was observed in the hospital-based controls for some cancer types, such as glioma. The reason may be that the hospital-based studies have some biases because such controls may contain certain benign diseases which are prone to develop malignancy and may not be very representative of the general population. Thus, the use of a proper and representative cancer-free control subjects is very important in reducing biases in such genotype association studies. Possible sources of heterogeneity, such as controls source, cancer type and ethnicity did not demonstrate the evidence of any significant variation by meta-regression. It is possible that other limitations of recruited studies may partially contribute to the observed heterogeneity. And this indicates that it may be not appropriate to use an overall estimation of the relationship between XRCC1 Arg399Gln polymorphism and risk of cancer.

The current meta-analysis has some strength compared with individual studies and previous meta-analyses. First, differently from previous meta-analyses [8]–[12], [25]–[35], we explored the impact of XRCC1 Arg399Gln on a great diversity of cancer sites, allowing for a general view of its influence on cancer susceptibility. Second, our meta-analysis explores and analyzes the sources of heterogeneity between studies about XRCC1 Arg399Gln in cancer. Third, more up to date studies were recruited to provide statistically significant results. As an example of these crucial features, differently from a recent pooled analysis of 23 studies in colorectal cancer [35] we found 27 studies in colorectal cancer. There was some evidence of publication bias for some cancers, such as breast cancer, which may also have contributed to the high heterogeneity observed. However, such limitations highlight the need for further studies in specific tumor sites and different ethnicities, using population-based sources of cases and controls and adequate sample size.

In summary, this meta-analysis suggests the participation of XRCC1 Arg399Gln is a genetic susceptibility for hepatocellular cancer in Asians and breast cancer in Indians. Moreover, our work also points out the importance of new studies for Arg399Gln association in some cancer types, such as glioma, gastric cancer, and oral cancer, where at least some of the covariates responsible for heterogeneity could be controlled, to obtain a more conclusive understanding about the function of the XRCC1 Arg399Gln polymorphism in cancer development.

## Supporting Information

References S1 References of Case-control studies included in the meta-analysis.
(DOC)

Table S1 Case-control studies included in the meta-analysis.
(DOC)
