# Supplementary material for: Association between the XRCC1 Arg399Gln Polymorphism and Risk of Cancer: Evidence from 297 Case–Control Studies
Source: PLoS One. 2013 Oct 29;8(10):e78071. doi: 10.1371/journal.pone.0078071 (PMC3812151; doi:10.1371/journal.pone.0078071)
Supplement: References S1 — References of Case-control studies included in the meta-analysis. (DOC) [file pone.0078071.s001.doc]

**References:**

1. Mohana DS, Balachandar V, Arun M, Suresh KS, Balamurali KB, et al. (2013) Analysis of genetic damage and gene polymorphism in hepatocellular carcinoma (HCC) patients in a South Indian population. Dig Dis Sci 58: 759-767.

2. Gulnaz A, Sayyed AH, Amin F, Khan A, Aslam MA, et al. (2013) Association of XRCC1, XRCC3, and XPD genetic polymorphism with an increased risk of hepatocellular carcinoma because of the hepatitis B and C virus. Eur J Gastroenterol Hepatol 25: 166-179.

3. Santos LS, Branco SC, Silva SN, Azevedo AP, Gil OM, et al. (2012) Polymorphisms in base excision repair genes and thyroid cancer risk. Oncol Rep 28: 1859-1868.

4. Muniz-Mendoza R, Ayala-Madrigal ML, Partida-Perez M, Peregrina-Sandoval J, Leal-Ugarte E, et al. (2012) MLH1 and XRCC1 polymorphisms in Mexican patients with colorectal cancer. Genet Mol Res 11: 2315-2320.

5. Abramenko I, Bilous N, Chumak A, Kostin A, Martina Z, et al. (2012) DNA repair polymorphisms in B-cell chronic lymphocytic leukemia in sufferers of Chernobyl Nuclear Power Plant accident. J Radiat Res 53: 497-503.

6. Farkasova T, Gurska S, Witkovsky V, Gabelova A (2008) Significance of amino acid substitution variants of DNA repair genes in radiosusceptibility of cervical cancer patients; a pilot study. Neoplasma 55: 330-337.

7. Chiyomaru K, Nagano T, Nishigori C (2012) XRCC1 Arg194Trp polymorphism, risk of nonmelanoma skin cancer and extramammary Paget's disease in a Japanese population. Arch Dermatol Res 304: 363-370.

8. Wang N, Wu Y, Zhou X, Wu Y (2012) [Association between genetic polymorphism of metabolizing enzymes and DNA repairing enzymes and the susceptibility of lung cancer in Henan population]. Wei Sheng Yan Jiu 41: 251-256.

9. Kostrzewska-Poczekaj M, Gawecki W, Illmer J, Rydzanicz M, Gajecka M, et al. (2013) Polymorphisms of DNA repair genes and risk of squamous cell carcinoma of the head and neck in young adults. Eur Arch Otorhinolaryngol 270: 271-276.

10. Zhou LQ, Ma Z, Shi XF, Yin XL, Huang KX, et al. (2011) Polymorphisms of DNA repair gene XRCC1 and risk of glioma: a case-control study in Southern China. Asian Pac J Cancer Prev 12: 2547-2550.

11. Sobczuk A, Poplawski T, Blasiak J (2012) Polymorphisms of DNA repair genes in endometrial cancer. Pathol Oncol Res 18: 1015-1020.

12. Yin G, Morita M, Ohnaka K, Toyomura K, Hamajima N, et al. (2012) Genetic polymorphisms of XRCC1, alcohol consumption, and the risk of colorectal cancer in Japan. J Epidemiol 22: 64-71.

13. Zhao Y, Deng X, Wang Z, Wang Q, Liu Y (2012) Genetic polymorphisms of DNA repair genes XRCC1 and XRCC3 and risk of colorectal cancer in Chinese population. Asian Pac J Cancer Prev 13: 665-669.

14. Duman N, Aktan M, Ozturk S, Palanduz S, Cakiris A, et al. (2012) Investigation of Arg399Gln and Arg194Trp polymorphisms of the XRCC1 (x-ray cross-complementing group 1) gene and its correlation to sister chromatid exchange frequency in patients with chronic lymphocytic leukemia. Genet Test Mol Biomarkers 16: 287-291.

15. Li QW, Lu CR, Ye M, Xiao WH, Liang J (2012) Evaluation of DNA repair gene XRCC1 polymorphism in prediction and prognosis of hepatocellular carcinoma risk. Asian Pac J Cancer Prev 13: 191-194.

16. Han X, Xing Q, Li Y, Sun J, Ji H, et al. (2012) Study on the DNA repair gene XRCC1 and XRCC3 polymorphism in prediction and prognosis of hepatocellular carcinoma risk. Hepatogastroenterology 59: 2285-2289.

17. Samulak D, Romanowicz-Makowska H, Smolarz B, Kulig A, Sporny S (2011) Association between Arg399Gln polymorphism of X-ray repair cross-complementing 1 (XRCC1) gene and sporadic endometrial cancer in the Polish population. Eur J Gynaecol Oncol 32: 491-495.

18. Mittal RD, Mandal RK, Gangwar R (2012) Base excision repair pathway genes polymorphism in prostate and bladder cancer risk in North Indian population. Mech Ageing Dev 133: 127-132.

19. Fard-Esfahani P, Fard-Esfahani A, Fayaz S, Ghanbarzadeh B, Saidi P, et al. (2011) Association of Arg194Trp, Arg280His and Arg399Gln polymorphisms in X-ray repair cross-complementing group 1 gene and risk of differentiated thyroid carcinoma in Iran. Iran Biomed J 15: 73-78.

20. Engin AB, Karahalil B, Karakaya AE, Engin A (2011) Association between XRCC1 ARG399GLN and P53 ARG72PRO polymorphisms and the risk of gastric and colorectal cancer in Turkish population. Arh Hig Rada Toksikol 62: 207-214.

21. Kumar A, Pant MC, Singh HS, Khandelwal S (2012) Associated risk of XRCC1 and XPD cross talk and life style factors in progression of head and neck cancer in north Indian population. Mutat Res 729: 24-34.

22. Roszak A, Lianeri M, Jagodzinski PP (2011) Involvement of the XRCC1 Arg399Gln gene polymorphism in the development of cervical carcinoma. Int J Biol Markers 26: 216-220.

23. Romanowicz-Makowska H, Smolarz B, Houli A, Szyllo K (2011) Single nucleotide polymorphism in DNA base excision repair genes XRCC1 and hOGG1 and the risk of endometrial carcinoma in the Polish population. Pol J Pathol 62: 89-94.

24. Ryu RA, Tae K, Min HJ, Jeong JH, Cho SH, et al. (2011) XRCC1 polymorphisms and risk of papillary thyroid carcinoma in a Korean sample. J Korean Med Sci 26: 991-995.

25. Barbisan G, Perez LO, Difranza L, Fernandez CJ, Ciancio NE, et al. (2011) XRCC1 Arg399Gln polymorphism and risk for cervical cancer development in Argentine women. Eur J Gynaecol Oncol 32: 274-279.

26. Settheetham-Ishida W, Yuenyao P, Natphopsuk S, Settheetham D, Ishida T (2011) Genetic risk of DNA repair gene polymorphisms (XRCC1 and XRCC3) for high risk human papillomavirus negative cervical cancer in Northeast Thailand. Asian Pac J Cancer Prev 12: 963-966.

27. Yu H, Fu C, Wang J, Xue H, Xu B (2011) Interaction between XRCC1 polymorphisms and intake of long-term stored rice in the risk of esophageal squamous cell carcinoma: a case-control study. Biomed Environ Sci 24: 268-274.

28. Li Z, Guan W, Li MX, Zhong ZY, Qian CY, et al. (2011) Genetic polymorphism of DNA base-excision repair genes (APE1, OGG1 and XRCC1) and their correlation with risk of lung cancer in a Chinese population. Arch Med Res 42: 226-234.

29. Berhane N, Sobti RC, Mahdi SA (2012) DNA repair genes polymorphism (XPG and XRCC1) and association of prostate cancer in a north Indian population. Mol Biol Rep 39: 2471-2479.

30. Hussien YM, Gharib AF, Awad HA, Karam RA, Elsawy WH (2012) Impact of DNA repair genes polymorphism (XPD and XRCC1) on the risk of breast cancer in Egyptian female patients. Mol Biol Rep 39: 1895-1901.

31. Santos RA, Teixeira AC, Mayorano MB, Carrara HH, Andrade JM, et al. (2010) DNA repair genes XRCC1 and XRCC3 polymorphisms and their relationship with the level of micronuclei in breast cancer patients. Genet Mol Biol 33: 637-640.

32. Roberts MR, Shields PG, Ambrosone CB, Nie J, Marian C, et al. (2011) Single-nucleotide polymorphisms in DNA repair genes and association with breast cancer risk in the web study. Carcinogenesis 32: 1223-1230.

33. Gsur A, Bernhart K, Baierl A, Feik E, Fuhrlinger G, et al. (2011) No association of XRCC1 polymorphisms Arg194Trp and Arg399Gln with colorectal cancer risk. Cancer Epidemiol 35: e38-e41.

34. Canbay E, Cakmakoglu B, Zeybek U, Sozen S, Cacina C, et al. (2011) Association of APE1 and hOGG1 polymorphisms with colorectal cancer risk in a Turkish population. Curr Med Res Opin 27: 1295-1302.

35. Gil J, Ramsey D, Stembalska A, Karpinski P, Pesz KA, et al. (2012) The C/A polymorphism in intron 11 of the XPC gene plays a crucial role in the modulation of an individual's susceptibility to sporadic colorectal cancer. Mol Biol Rep 39: 527-534.

36. Chen DJ, Ding R, Cao W, Ye DQ (2011) [Interaction between polymorphisms in NQO1(C609T) and XRCC1(G28152A) and their correlation with smoking on gastric cancer]. Zhonghua Liu Xing Bing Xue Za Zhi 32: 5-8.

37. Bianchino G, Cittadini A, Grieco V, Traficante A, Zupa A, et al. (2011) Polymorphisms of the CYP1A1, CYP2E1 and XRCC1 genes and cancer risk in a Southern Italian population: a case-control study. Anticancer Res 31: 1359-1365.

38. Canalle R, Silveira VS, Scrideli CA, Queiroz RG, Lopes LF, et al. (2011) Impact of thymidylate synthase promoter and DNA repair gene polymorphisms on susceptibility to childhood acute lymphoblastic leukemia. Leuk Lymphoma 52: 1118-1126.

39. Krupa R, Kasznicki J, Gajecka M, Rydzanicz M, Kiwerska K, et al. (2011) Polymorphisms of the DNA repair genes XRCC1 and ERCC4 are not associated with smoking- and drinking-dependent larynx cancer in a Polish population. Exp Oncol 33: 55-56.

40. Gugatschka M, Dehchamani D, Wascher TC, Friedrich G, Renner W (2011) DNA repair gene ERCC2 polymorphisms and risk of squamous cell carcinoma of the head and neck. Exp Mol Pathol 91: 331-334.

41. Garcia-Quispes WA, Perez-Machado G, Akdi A, Pastor S, Galofre P, et al. (2011) Association studies of OGG1, XRCC1, XRCC2 and XRCC3 polymorphisms with differentiated thyroid cancer. Mutat Res 709-710: 67-72.

42. Janik J, Swoboda M, Janowska B, Ciesla JM, Gackowski D, et al. (2011) 8-Oxoguanine incision activity is impaired in lung tissues of NSCLC patients with the polymorphism of OGG1 and XRCC1 genes. Mutat Res 709-710: 21-31.

43. Monroy CM, Cortes AC, Lopez M, Rourke E, Etzel CJ, et al. (2011) Hodgkin lymphoma risk: role of genetic polymorphisms and gene-gene interactions in DNA repair pathways. Mol Carcinog 50: 825-834.

44. Romanowicz H, Smolarz B, Baszczynski J, Zadrozny M, Kulig A (2010) Genetics polymorphism in DNA repair genes by base excision repair pathway (XRCC1) and homologous recombination (XRCC2 and RAD51) and the risk of breast carcinoma in the Polish population. Pol J Pathol 61: 206-212.

45. Betti M, Ferrante D, Padoan M, Guarrera S, Giordano M, et al. (2011) XRCC1 and ERCC1 variants modify malignant mesothelioma risk: a case-control study. Mutat Res 708: 11-20.

46. Osawa K, Miyaishi A, Uchino K, Osawa Y, Inoue N, et al. (2010) APEX1 Asp148Glu gene polymorphism is a risk factor for lung cancer in relation to smoking in Japanese. Asian Pac J Cancer Prev 11: 1181-1186.

47. Qian B, Zhang H, Zhang L, Zhou X, Yu H, et al. (2011) Association of genetic polymorphisms in DNA repair pathway genes with non-small cell lung cancer risk. Lung Cancer 73: 138-146.

48. Pan HZ, Liang J, Yu Z, Lun LM, Li H, et al. (2011) Polymorphism of DNA repair gene XRCC1 and hepatocellular carcinoma risk in Chinese population. Asian Pac J Cancer Prev 12: 2947-2950.

49. Brevik A, Joshi AD, Corral R, Onland-Moret NC, Siegmund KD, et al. (2010) Polymorphisms in base excision repair genes as colorectal cancer risk factors and modifiers of the effect of diets high in red meat. Cancer Epidemiol Biomarkers Prev 19: 3167-3173.

50. Kim IS, Lee GW, Kim DC, Kim HG, Kim S, et al. (2010) Polymorphisms and haplotypes in the XRCC1 gene and the risk of advanced non-small cell lung cancer. J Thorac Oncol 5: 1912-1921.

51. Yosunkaya E, Kucukyuruk B, Onaran I, Gurel CB, Uzan M, et al. (2010) Glioma risk associates with polymorphisms of DNA repair genes, XRCC1 and PARP1. Br J Neurosurg 24: 561-565.

52. Kuasne H, Rodrigues IS, Losi-Guembarovski R, Reis MB, Fuganti PE, et al. (2011) Base excision repair genes XRCC1 and APEX1 and the risk for prostate cancer. Mol Biol Rep 38: 1585-1591.

53. Palli D, Polidoro S, D'Errico M, Saieva C, Guarrera S, et al. (2010) Polymorphic DNA repair and metabolic genes: a multigenic study on gastric cancer. Mutagenesis 25: 569-575.

54. Jelonek K, Gdowicz-Klosok A, Pietrowska M, Borkowska M, Korfanty J, et al. (2010) Association between single-nucleotide polymorphisms of selected genes involved in the response to DNA damage and risk of colon, head and neck, and breast cancers in a Polish population. J Appl Genet 51: 343-352.

55. Liu L, Yuan P, Liu L, Wu C, Zhang X, et al. (2011) A functional -77T>C polymorphism in XRCC1 is associated with risk of breast cancer. Breast Cancer Res Treat 125: 479-487.

56. Canbay E, Agachan B, Gulluoglu M, Isbir T, Balik E, et al. (2010) Possible associations of APE1 polymorphism with susceptibility and HOGG1 polymorphism with prognosis in gastric cancer. Anticancer Res 30: 1359-1364.

57. Gao W, Romkes M, Zhong S, Nukui T, Persad RA, et al. (2010) Genetic polymorphisms in the DNA repair genes XPD and XRCC1, p53 gene mutations and bladder cancer risk. Oncol Rep 24: 257-262.

58. Gao R, Price DK, Dahut WL, Reed E, Figg WD (2010) Genetic polymorphisms in XRCC1 associated with radiation therapy in prostate cancer. Cancer Biol Ther 10: 13-18.

59. Qian Y, Zhang JP, Dong J, Wang FR, Lin YD, et al. (2010) [Relationship between polymorphisms of X-ray repair cross-complementing group 1 gene Arg194Trp, Arg399Gln and susceptibility of breast cancer]. Zhonghua Yu Fang Yi Xue Za Zhi 44: 242-246.

60. Zipprich J, Terry MB, Brandt-Rauf P, Freyer GA, Liao Y, et al. (2010) XRCC1 polymorphisms and breast cancer risk from the New York Site of the Breast Cancer Family Registry: A family-based case-control study. J Carcinog 9: 4.

61. Tumer TB, Yilmaz D, Tanrikut C, Sahin G, Ulusoy G, et al. (2010) DNA repair XRCC1 Arg399Gln polymorphism alone, and in combination with CYP2E1 polymorphisms significantly contribute to the risk of development of childhood acute lymphoblastic leukemia. Leuk Res 34: 1275-1281.

62. Stanczyk M, Sliwinski T, Cuchra M, Zubowska M, Bielecka-Kowalska A, et al. (2011) The association of polymorphisms in DNA base excision repair genes XRCC1, OGG1 and MUTYH with the risk of childhood acute lymphoblastic leukemia. Mol Biol Rep 38: 445-451.

63. Alsbeih G, Al-Harbi N, Al-Hadyan K, El-Sebaie M, Al-Rajhi N (2010) Association between normal tissue complications after radiotherapy and polymorphic variations in TGFB1 and XRCC1 genes. Radiat Res 173: 505-511.

64. Wang J, Zhao Y, Jiang J, Gajalakshmi V, Kuriki K, et al. (2010) Polymorphisms in DNA repair genes XRCC1, XRCC3 and XPD, and colorectal cancer risk: a case-control study in an Indian population. J Cancer Res Clin Oncol 136: 1517-1525.

65. Wang M, Qin C, Zhu J, Yuan L, Fu G, et al. (2010) Genetic variants of XRCC1, APE1, and ADPRT genes and risk of bladder cancer. DNA Cell Biol 29: 303-311.

66. Ming-Shiean H, Yu JC, Wang HW, Chen ST, Hsiung CN, et al. (2010) Synergistic effects of polymorphisms in DNA repair genes and endogenous estrogen exposure on female breast cancer risk. Ann Surg Oncol 17: 760-771.

67. Rajaraman P, Hutchinson A, Wichner S, Black PM, Fine HA, et al. (2010) DNA repair gene polymorphisms and risk of adult meningioma, glioma, and acoustic neuroma. Neuro Oncol 12: 37-48.

68. Sterpone S, Mastellone V, Padua L, Novelli F, Patrono C, et al. (2010) Single-nucleotide polymorphisms in BER and HRR genes, XRCC1 haplotypes and breast cancer risk in Caucasian women. J Cancer Res Clin Oncol 136: 631-636.

69. Mandal RK, Gangwar R, Mandhani A, Mittal RD (2010) DNA repair gene X-ray repair cross-complementing group 1 and xeroderma pigmentosum group D polymorphisms and risk of prostate cancer: a study from North India. DNA Cell Biol 29: 183-190.

70. Bhattacharyya N, Banerjee S (2001) A novel role of XRCC1 in the functions of a DNA polymerase beta variant. Biochemistry 40: 9005-9013.

71. Meza-Espinoza JP, Peralta-Leal V, Gutierrez-Angulo M, Macias-Gomez N, Ayala-Madrigal ML, et al. (2009) XRCC1 polymorphisms and haplotypes in Mexican patients with acute lymphoblastic leukemia. Genet Mol Res 8: 1451-1458.

72. Kim IS, Kim DC, Kim HG, Eom HS, Kong SY, et al. (2010) DNA repair gene XRCC1 polymorphisms and haplotypes in diffuse large B-cell lymphoma in a Korean population. Cancer Genet Cytogenet 196: 31-37.

73. Han J, Hankinson SE, De Vivo I, Spiegelman D, Tamimi RM, et al. (2003) A prospective study of XRCC1 haplotypes and their interaction with plasma carotenoids on breast cancer risk. Cancer Res 63: 8536-8541.

74. Curtin K, Samowitz WS, Wolff RK, Ulrich CM, Caan BJ, et al. (2009) Assessing tumor mutations to gain insight into base excision repair sequence polymorphisms and smoking in colon cancer. Cancer Epidemiol Biomarkers Prev 18: 3384-3388.

75. Zhang J, Dhakal IB, Greene G, Lang NP, Kadlubar FF (2010) Polymorphisms in hOGG1 and XRCC1 and risk of prostate cancer: effects modified by plasma antioxidants. Urology 75: 779-785.

76. Dhillon VS, Yeoh E, Fenech M (2011) DNA repair gene polymorphisms and prostate cancer risk in South Australia--results of a pilot study. Urol Oncol 29: 641-646.

77. Agalliu I, Kwon EM, Salinas CA, Koopmeiners JS, Ostrander EA, et al. (2010) Genetic variation in DNA repair genes and prostate cancer risk: results from a population-based study. Cancer Causes Control 21: 289-300.

78. Sobczuk A, Romanowicz-Makowska H, Fiks T, Baszczynski J, Smolarz B (2009) XRCC1 and XRCC3 DNA repair gene polymorphisms in breast cancer women from the Lodz region of Poland. Pol J Pathol 60: 76-80.

79. Csejtei A, Tibold A, Koltai K, Varga Z, Szanyi I, et al. (2009) Association between XRCC1 polymorphisms and head and neck cancer in a Hungarian population. Anticancer Res 29: 4169-4173.

80. Duell EJ, Millikan RC, Pittman GS, Winkel S, Lunn RM, et al. (2001) Polymorphisms in the DNA repair gene XRCC1 and breast cancer. Cancer Epidemiol Biomarkers Prev 10: 217-222.

81. Yan L, Yanan D, Donglan S, Na W, Rongmiao Z, et al. (2009) Polymorphisms of XRCC1 gene and risk of gastric cardiac adenocarcinoma. Dis Esophagus 22: 396-401.

82. Baris S, Celkan T, Batar B, Guven M, Ozdil M, et al. (2009) Association between genetic polymorphism in DNA repair genes and risk of B-cell lymphoma. Pediatr Hematol Oncol 26: 467-472.

83. Ganster C, Neesen J, Zehetmayer S, Jager U, Esterbauer H, et al. (2009) DNA repair polymorphisms associated with cytogenetic subgroups in B-cell chronic lymphocytic leukemia. Genes Chromosomes Cancer 48: 760-767.

84. Liu J, Song B, Wang Z, Song X, Shi Y, et al. (2009) DNA repair gene XRCC1 polymorphisms and non-Hodgkin lymphoma risk in a Chinese population. Cancer Genet Cytogenet 191: 67-72.

85. Lee JM, Lee YC, Yang SY, Yang PW, Luh SP, et al. (2001) Genetic polymorphisms of XRCC1 and risk of the esophageal cancer. Int J Cancer 95: 240-246.

86. Wen H, Ding Q, Fang ZJ, Xia GW, Fang J (2009) Population study of genetic polymorphisms and superficial bladder cancer risk in Han-Chinese smokers in Shanghai. Int Urol Nephrol 41: 855-864.

87. Varzim G, Monteiro E, Silva RA, Fernandes J, Lopes C (2003) CYP1A1 and XRCC1 gene polymorphisms in SCC of the larynx. Eur J Cancer Prev 12: 495-499.

88. Akulevich NM, Saenko VA, Rogounovitch TI, Drozd VM, Lushnikov EF, et al. (2009) Polymorphisms of DNA damage response genes in radiation-related and sporadic papillary thyroid carcinoma. Endocr Relat Cancer 16: 491-503.

89. Kowalski M, Przybylowska K, Rusin P, Olszewski J, Morawiec-Sztandera A, et al. (2009) Genetic polymorphisms in DNA base excision repair gene XRCC1 and the risk of squamous cell carcinoma of the head and neck. J Exp Clin Cancer Res 28: 37.

90. El-Zein R, Monroy CM, Etzel CJ, Cortes AC, Xing Y, et al. (2009) Genetic polymorphisms in DNA repair genes as modulators of Hodgkin disease risk. Cancer 115: 1651-1659.

91. Srivastava A, Srivastava K, Pandey SN, Choudhuri G, Mittal B (2009) Single-nucleotide polymorphisms of DNA repair genes OGG1 and XRCC1: association with gallbladder cancer in North Indian population. Ann Surg Oncol 16: 1695-1703.

92. Applebaum KM, McClean MD, Nelson HH, Marsit CJ, Christensen BC, et al. (2009) Smoking modifies the relationship between XRCC1 haplotypes and HPV16-negative head and neck squamous cell carcinoma. Int J Cancer 124: 2690-2696.

93. Kiran M, Saxena R, Chawla YK, Kaur J (2009) Polymorphism of DNA repair gene XRCC1 and hepatitis-related hepatocellular carcinoma risk in Indian population. Mol Cell Biochem 327: 7-13.

94. Ho T, Li G, Lu J, Zhao C, Wei Q, et al. (2009) Association of XRCC1 polymorphisms and risk of differentiated thyroid carcinoma: a case-control analysis. Thyroid 19: 129-135.

95. Cote ML, Yoo W, Wenzlaff AS, Prysak GM, Santer SK, et al. (2009) Tobacco and estrogen metabolic polymorphisms and risk of non-small cell lung cancer in women. Carcinogenesis 30: 626-635.

96. Liu Y, Scheurer ME, El-Zein R, Cao Y, Do KA, et al. (2009) Association and interactions between DNA repair gene polymorphisms and adult glioma. Cancer Epidemiol Biomarkers Prev 18: 204-214.

97. Batar B, Guven M, Baris S, Celkan T, Yildiz I (2009) DNA repair gene XPD and XRCC1 polymorphisms and the risk of childhood acute lymphoblastic leukemia. Leuk Res 33: 759-763.

98. Siraj AK, Al-Rasheed M, Ibrahim M, Siddiqui K, Al-Dayel F, et al. (2008) RAD52 polymorphisms contribute to the development of papillary thyroid cancer susceptibility in Middle Eastern population. J Endocrinol Invest 31: 893-899.

99. Yin J, Vogel U, Ma Y, Qi R, Wang H (2009) Association of DNA repair gene XRCC1 and lung cancer susceptibility among nonsmoking Chinese women. Cancer Genet Cytogenet 188: 26-31.

100. Syamala VS, Syamala V, Sreedharan H, Raveendran PB, Kuttan R, et al. (2009) Contribution of XPD (Lys751Gln) and XRCC1 (Arg399Gln) polymorphisms in familial and sporadic breast cancer predisposition and survival: an Indian report. Pathol Oncol Res 15: 389-397.

101. Improta G, Sgambato A, Bianchino G, Zupa A, Grieco V, et al. (2008) Polymorphisms of the DNA repair genes XRCC1 and XRCC3 and risk of lung and colorectal cancer: a case-control study in a Southern Italian population. Anticancer Res 28: 2941-2946.

102. Hung RJ, Christiani DC, Risch A, Popanda O, Haugen A, et al. (2008) International Lung Cancer Consortium: pooled analysis of sequence variants in DNA repair and cell cycle pathways. Cancer Epidemiol Biomarkers Prev 17: 3081-3089.

103. Kasahara M, Osawa K, Yoshida K, Miyaishi A, Osawa Y, et al. (2008) Association of MUTYH Gln324His and APEX1 Asp148Glu with colorectal cancer and smoking in a Japanese population. J Exp Clin Cancer Res 27: 49.

104. Sliwinski T, Krupa R, Wisniewska-Jarosinska M, Lech J, Morawiec Z, et al. (2008) No association between the Arg194Trp and Arg399Gln polymorphisms of the XRCC1 gene and colorectal cancer risk and progression in a Polish population. Exp Oncol 30: 253-254.

105. Chiang FY, Wu CW, Hsiao PJ, Kuo WR, Lee KW, et al. (2008) Association between polymorphisms in DNA base excision repair genes XRCC1, APE1, and ADPRT and differentiated thyroid carcinoma. Clin Cancer Res 14: 5919-5924.

106. Alsbeih GA, El-Sebaie MM, Al-Rajhi NM, Al-Harbi NM, Al-Hadyan KS, et al. (2008) Association between XRCC1 G399A Polymorphism and Late Complications to Radiotherapy in Saudi Head and Neck Cancer Patients. J Egypt Natl Canc Inst 20: 302-308.

107. Smith TR, Levine EA, Freimanis RI, Akman SA, Allen GO, et al. (2008) Polygenic model of DNA repair genetic polymorphisms in human breast cancer risk. Carcinogenesis 29: 2132-2138.

108. Mitra AK, Singh N, Singh A, Garg VK, Agarwal A, et al. (2008) Association of polymorphisms in base excision repair genes with the risk of breast cancer: a case-control study in North Indian women. Oncol Res 17: 127-135.

109. Capella G, Pera G, Sala N, Agudo A, Rico F, et al. (2008) DNA repair polymorphisms and the risk of stomach adenocarcinoma and severe chronic gastritis in the EPIC-EURGAST study. Int J Epidemiol 37: 1316-1325.

110. Fontana L, Bosviel R, Delort L, Guy L, Chalabi N, et al. (2008) DNA repair gene ERCC2, XPC, XRCC1, XRCC3 polymorphisms and associations with bladder cancer risk in a French cohort. Anticancer Res 28: 1853-1856.

111. Harth V, Schafer M, Abel J, Maintz L, Neuhaus T, et al. (2008) Head and neck squamous-cell cancer and its association with polymorphic enzymes of xenobiotic metabolism and repair. J Toxicol Environ Health A 71: 887-897.

112. McWilliams RR, Bamlet WR, Cunningham JM, Goode EL, de Andrade M, et al. (2008) Polymorphisms in DNA repair genes, smoking, and pancreatic adenocarcinoma risk. Cancer Res 68: 4928-4935.

113. Tse D, Zhai R, Zhou W, Heist RS, Asomaning K, et al. (2008) Polymorphisms of the NER pathway genes, ERCC1 and XPD are associated with esophageal adenocarcinoma risk. Cancer Causes Control 19: 1077-1083.

114. Kipikasova L, Wolaschka T, Bohus P, Baumohlova H, Bober J, et al. (2008) Polymorphisms of the XRCC1 and XPD genes and breast cancer risk: a case-control study. Pathol Oncol Res 14: 131-135.

115. Li M, Yin Z, Guan P, Li X, Cui Z, et al. (2008) XRCC1 polymorphisms, cooking oil fume and lung cancer in Chinese women nonsmokers. Lung Cancer 62: 145-151.

116. Yang Y, Tian H, Zhang ZJ (2008) [Association of the XRCC1 and hOGG1 polymorphisms with the risk of laryngeal carcinoma]. Zhonghua Yi Xue Yi Chuan Xue Za Zhi 25: 211-213.

117. Doecke J, Zhao ZZ, Pandeya N, Sadeghi S, Stark M, et al. (2008) Polymorphisms in MGMT and DNA repair genes and the risk of esophageal adenocarcinoma. Int J Cancer 123: 174-180.

118. Covolo L, Placidi D, Gelatti U, Carta A, Scotto DCA, et al. (2008) Bladder cancer, GSTs, NAT1, NAT2, SULT1A1, XRCC1, XRCC3, XPD genetic polymorphisms and coffee consumption: a case-control study. Eur J Epidemiol 23: 355-362.

119. Mittal RD, Singh R, Manchanda PK, Ahirwar D, Gangwar R, et al. (2008) XRCC1 codon 399 mutant allele: a risk factor for recurrence of urothelial bladder carcinoma in patients on BCG immunotherapy. Cancer Biol Ther 7: 645-650.

120. Ali MF, Meza JL, Rogan EG, Chakravarti D (2008) Prevalence of BER gene polymorphisms in sporadic breast cancer. Oncol Rep 19: 1033-1038.

121. Ferguson HR, Wild CP, Anderson LA, Murphy SJ, Johnston BT, et al. (2008) No association between hOGG1, XRCC1, and XPD polymorphisms and risk of reflux esophagitis, Barrett's esophagus, or esophageal adenocarcinoma: results from the factors influencing the Barrett's adenocarcinoma relationship case-control study. Cancer Epidemiol Biomarkers Prev 17: 736-739.

122. Kiuru A, Lindholm C, Heinavaara S, Ilus T, Jokinen P, et al. (2008) XRCC1 and XRCC3 variants and risk of glioma and meningioma. J Neurooncol 88: 135-142.

123. Arizono K, Osada Y, Kuroda Y (2008) DNA repair gene hOGG1 codon 326 and XRCC1 codon 399 polymorphisms and bladder cancer risk in a Japanese population. Jpn J Clin Oncol 38: 186-191.

124. Hsu LI, Chiu AW, Huan SK, Chen CL, Wang YH, et al. (2008) SNPs of GSTM1, T1, P1, epoxide hydrolase and DNA repair enzyme XRCC1 and risk of urinary transitional cell carcinoma in southwestern Taiwan. Toxicol Appl Pharmacol 228: 144-155.

125. Loizidou MA, Michael T, Neuhausen SL, Newbold RF, Marcou Y, et al. (2008) Genetic polymorphisms in the DNA repair genes XRCC1, XRCC2 and XRCC3 and risk of breast cancer in Cyprus. Breast Cancer Res Treat 112: 575-579.

126. Stern MC, Conti DV, Siegmund KD, Corral R, Yuan JM, et al. (2007) DNA repair single-nucleotide polymorphisms in colorectal cancer and their role as modifiers of the effect of cigarette smoking and alcohol in the Singapore Chinese Health Study. Cancer Epidemiol Biomarkers Prev 16: 2363-2372.

127. Saadat M, Kohan L, Omidvari S (2008) Genetic polymorphisms of XRCC1 (codon 399) and susceptibility to breast cancer in Iranian women, a case-control study. Breast Cancer Res Treat 111: 549-553.

128. Huang WY, Gao YT, Rashid A, Sakoda LC, Deng J, et al. (2008) Selected base excision repair gene polymorphisms and susceptibility to biliary tract cancer and biliary stones: a population-based case-control study in China. Carcinogenesis 29: 100-105.

129. Sreeja L, Syamala VS, Syamala V, Hariharan S, Raveendran PB, et al. (2008) Prognostic importance of DNA repair gene polymorphisms of XRCC1 Arg399Gln and XPD Lys751Gln in lung cancer patients from India. J Cancer Res Clin Oncol 134: 645-652.

130. Silva SN, Moita R, Azevedo AP, Gouveia R, Manita I, et al. (2007) Menopausal age and XRCC1 gene polymorphisms: role in breast cancer risk. Cancer Detect Prev 31: 303-309.

131. Sangrajrang S, Schmezer P, Burkholder I, Waas P, Boffetta P, et al. (2008) Polymorphisms in three base excision repair genes and breast cancer risk in Thai women. Breast Cancer Res Treat 111: 279-288.

132. Andrew AS, Karagas MR, Nelson HH, Guarrera S, Polidoro S, et al. (2008) DNA repair polymorphisms modify bladder cancer risk: a multi-factor analytic strategy. Hum Hered 65: 105-118.

133. Felini MJ, Olshan AF, Schroeder JC, North KE, Carozza SE, et al. (2007) DNA repair polymorphisms XRCC1 and MGMT and risk of adult gliomas. Neuroepidemiology 29: 55-58.

134. Borentain P, Gerolami V, Ananian P, Garcia S, Noundou A, et al. (2007) DNA-repair and carcinogen-metabolising enzymes genetic polymorphisms as an independent risk factor for hepatocellular carcinoma in Caucasian liver-transplanted patients. Eur J Cancer 43: 2479-2486.

135. Lopez-Cima MF, Gonzalez-Arriaga P, Garcia-Castro L, Pascual T, Marron MG, et al. (2007) Polymorphisms in XPC, XPD, XRCC1, and XRCC3 DNA repair genes and lung cancer risk in a population of northern Spain. BMC Cancer 7: 162.

136. Deligezer U, Akisik EE, Dalay N (2007) Lack of association of XRCC1 codon 399Gln polymorphism with chronic myelogenous leukemia. Anticancer Res 27: 2453-2456.

137. Yang ZH, Du B, Wei YS, Zhang JH, Zhou B, et al. (2007) Genetic polymorphisms of the DNA repair gene and risk of nasopharyngeal carcinoma. DNA Cell Biol 26: 491-496.

138. Ruzzo A, Canestrari E, Maltese P, Pizzagalli F, Graziano F, et al. (2007) Polymorphisms in genes involved in DNA repair and metabolism of xenobiotics in individual susceptibility to sporadic diffuse gastric cancer. Clin Chem Lab Med 45: 822-828.

139. Li C, Hu Z, Lu J, Liu Z, Wang LE, et al. (2007) Genetic polymorphisms in DNA base-excision repair genes ADPRT, XRCC1, and APE1 and the risk of squamous cell carcinoma of the head and neck. Cancer 110: 867-875.

140. Ho T, Li G, Lu J, Zhao C, Wei Q, et al. (2007) X-ray repair cross-complementing group 1 (XRCC1) single-nucleotide polymorphisms and the risk of salivary gland carcinomas. Cancer 110: 318-325.

141. Sobti RC, Singh J, Kaur P, Pachouri SS, Siddiqui EA, et al. (2007) XRCC1 codon 399 and ERCC2 codon 751 polymorphism, smoking, and drinking and risk of esophageal squamous cell carcinoma in a North Indian population. Cancer Genet Cytogenet 175: 91-97.

142. De Ruyck K, Szaumkessel M, De Rudder I, Dehoorne A, Vral A, et al. (2007) Polymorphisms in base-excision repair and nucleotide-excision repair genes in relation to lung cancer risk. Mutat Res 631: 101-110.

143. Huang J, Ye F, Chen H, Lu W, Xie X (2007) The nonsynonymous single nucleotide polymorphisms of DNA repair gene XRCC1 and susceptibility to the development of cervical carcinoma and high-risk human papillomavirus infection. Int J Gynecol Cancer 17: 668-675.

144. Xu Z, Qian LX, Hua LX, Wang XR, Yang J, et al. (2007) [Relationship between DNA repair gene XRCC1 Arg399Gln polymorphism and susceptibility to prostate cancer in the Han population in Jiangsu and Anhui]. Zhonghua Nan Ke Xue 13: 327-331.

145. Xu Z, Hua LX, Qian LX, Yang J, Wang XR, et al. (2007) Relationship between XRCC1 polymorphisms and susceptibility to prostate cancer in men from Han, Southern China. Asian J Androl 9: 331-338.

146. Sak SC, Barrett JH, Paul AB, Bishop DT, Kiltie AE (2007) DNA repair gene XRCC1 polymorphisms and bladder cancer risk. BMC Genet 8: 13.

147. Pachouri SS, Sobti RC, Kaur P, Singh J (2007) Contrasting impact of DNA repair gene XRCC1 polymorphisms Arg399Gln and Arg194Trp on the risk of lung cancer in the north-Indian population. DNA Cell Biol 26: 186-191.

148. Jin MJ, Chen K, Zhang Y, Zhang W, Liu B, et al. (2007) [Correlations of single nucleotide polymorphisms of DNA repair gene XRCC1 to risk of colorectal cancer]. Ai Zheng 26: 274-279.

149. Kang SY, Lee KG, Lee W, Shim JY, Ji SI, et al. (2007) Polymorphisms in the DNA repair gene XRCC1 associated with basal cell carcinoma and squamous cell carcinoma of the skin in a Korean population. Cancer Sci 98: 716-720.

150. Yin J, Vogel U, Ma Y, Qi R, Sun Z, et al. (2007) The DNA repair gene XRCC1 and genetic susceptibility of lung cancer in a northeastern Chinese population. Lung Cancer 56: 153-160.

151. Majumder M, Sikdar N, Ghosh S, Roy B (2007) Polymorphisms at XPD and XRCC1 DNA repair loci and increased risk of oral leukoplakia and cancer among NAT2 slow acetylators. Int J Cancer 120: 2148-2156.

152. Berndt SI, Huang WY, Fallin MD, Helzlsouer KJ, Platz EA, et al. (2007) Genetic variation in base excision repair genes and the prevalence of advanced colorectal adenoma. Cancer Res 67: 1395-1404.

153. Liu G, Zhou W, Yeap BY, Su L, Wain JC, et al. (2007) XRCC1 and XPD polymorphisms and esophageal adenocarcinoma risk. Carcinogenesis 28: 1254-1258.

154. Karahalil B, Kocabas NA, Ozcelik T (2006) DNA repair gene polymorphisms and bladder cancer susceptibility in a Turkish population. Anticancer Res 26: 4955-4958.

155. Figueroa JD, Malats N, Real FX, Silverman D, Kogevinas M, et al. (2007) Genetic variation in the base excision repair pathway and bladder cancer risk. Hum Genet 121: 233-242.

156. Hirata H, Hinoda Y, Tanaka Y, Okayama N, Suehiro Y, et al. (2007) Polymorphisms of DNA repair genes are risk factors for prostate cancer. Eur J Cancer 43: 231-237.

157. Yeh CC, Sung FC, Tang R, Chang-Chieh CR, Hsieh LL (2007) Association between polymorphisms of biotransformation and DNA-repair genes and risk of colorectal cancer in Taiwan. J Biomed Sci 14: 183-193.

158. Wang L, Lin DX, Lu XH, Miao XP, Li H (2006) [Polymorphisms of the DNA repair genes XRCC1 and XPC: relationship to pancreatic cancer risk]. Wei Sheng Yan Jiu 35: 534-536.

159. Costa S, Pinto D, Pereira D, Rodrigues H, Cameselle-Teijeiro J, et al. (2007) DNA repair polymorphisms might contribute differentially on familial and sporadic breast cancer susceptibility: a study on a Portuguese population. Breast Cancer Res Treat 103: 209-217.

160. Ryk C, Kumar R, Thirumaran RK, Hou SM (2006) Polymorphisms in the DNA repair genes XRCC1, APEX1, XRCC3 and NBS1, and the risk for lung cancer in never- and ever-smokers. Lung Cancer 54: 285-292.

161. (2006) Commonly studied single-nucleotide polymorphisms and breast cancer: results from the Breast Cancer Association Consortium. J Natl Cancer Inst 98: 1382-1396.

162. Thyagarajan B, Anderson KE, Folsom AR, Jacobs DJ, Lynch CF, et al. (2006) No association between XRCC1 and XRCC3 gene polymorphisms and breast cancer risk: Iowa Women's Health Study. Cancer Detect Prev 30: 313-321.

163. Jin MJ, Chen K, Zhang SS, Zhang YJ, Ren YJ, et al. (2006) [Association of single nucleotide polymorphisms and haplotypes in DNA repair gene XRCC1 with susceptibility of breast cancer]. Zhejiang Da Xue Xue Bao Yi Xue Ban 35: 370-376.

164. Miao X, Zhang X, Zhang L, Guo Y, Hao B, et al. (2006) Adenosine diphosphate ribosyl transferase and x-ray repair cross-complementing 1 polymorphisms in gastric cardia cancer. Gastroenterology 131: 420-427.

165. Long XD, Ma Y, Wei YP, Deng ZL (2006) The polymorphisms of GSTM1, GSTT1, HYL1*2, and XRCC1, and aflatoxin B1-related hepatocellular carcinoma in Guangxi population, China. Hepatol Res 36: 48-55.

166. Jiao L, Bondy ML, Hassan MM, Wolff RA, Evans DB, et al. (2006) Selected polymorphisms of DNA repair genes and risk of pancreatic cancer. Cancer Detect Prev 30: 284-291.

167. Cao Y, Miao XP, Huang MY, Deng L, Hu LF, et al. (2006) Polymorphisms of XRCC1 genes and risk of nasopharyngeal carcinoma in the Cantonese population. BMC Cancer 6: 167.

168. Hao B, Miao X, Li Y, Zhang X, Sun T, et al. (2006) A novel T-77C polymorphism in DNA repair gene XRCC1 contributes to diminished promoter activity and increased risk of non-small cell lung cancer. Oncogene 25: 3613-3620.

169. Cai L, You NC, Lu H, Mu LN, Lu QY, et al. (2006) Dietary selenium intake, aldehyde dehydrogenase-2 and X-ray repair cross-complementing 1 genetic polymorphisms, and the risk of esophageal squamous cell carcinoma. Cancer 106: 2345-2354.

170. Li C, Liu Z, Wang LE, Strom SS, Lee JE, et al. (2006) Genetic variants of the ADPRT, XRCC1 and APE1 genes and risk of cutaneous melanoma. Carcinogenesis 27: 1894-1901.

171. Moreno V, Gemignani F, Landi S, Gioia-Patricola L, Chabrier A, et al. (2006) Polymorphisms in genes of nucleotide and base excision repair: risk and prognosis of colorectal cancer. Clin Cancer Res 12: 2101-2108.

172. Bu D, Tomlinson G, Lewis CM, Zhang C, Kildebeck E, et al. (2006) An intronic polymorphism associated with increased XRCC1 expression, reduced apoptosis and familial breast cancer. Breast Cancer Res Treat 99: 257-265.

173. Ye W, Kumar R, Bacova G, Lagergren J, Hemminki K, et al. (2006) The XPD 751Gln allele is associated with an increased risk for esophageal adenocarcinoma: a population-based case-control study in Sweden. Carcinogenesis 27: 1835-1841.

174. Dianzani I, Gibello L, Biava A, Giordano M, Bertolotti M, et al. (2006) Polymorphisms in DNA repair genes as risk factors for asbestos-related malignant mesothelioma in a general population study. Mutat Res 599: 124-134.

175. Skjelbred CF, Saebo M, Wallin H, Nexo BA, Hagen PC, et al. (2006) Polymorphisms of the XRCC1, XRCC3 and XPD genes and risk of colorectal adenoma and carcinoma, in a Norwegian cohort: a case control study. BMC Cancer 6: 67.

176. Pachkowski BF, Winkel S, Kubota Y, Swenberg JA, Millikan RC, et al. (2006) XRCC1 genotype and breast cancer: functional studies and epidemiologic data show interactions between XRCC1 codon 280 His and smoking. Cancer Res 66: 2860-2868.

177. Hirata H, Hinoda Y, Matsuyama H, Tanaka Y, Okayama N, et al. (2006) Polymorphisms of DNA repair genes are associated with renal cell carcinoma. Biochem Biophys Res Commun 342: 1058-1062.

178. Zhang Y, Newcomb PA, Egan KM, Titus-Ernstoff L, Chanock S, et al. (2006) Genetic polymorphisms in base-excision repair pathway genes and risk of breast cancer. Cancer Epidemiol Biomarkers Prev 15: 353-358.

179. Smedby KE, Lindgren CM, Hjalgrim H, Humphreys K, Schollkopf C, et al. (2006) Variation in DNA repair genes ERCC2, XRCC1, and XRCC3 and risk of follicular lymphoma. Cancer Epidemiol Biomarkers Prev 15: 258-265.

180. Li MC, Cui ZS, He QC, Zhou BS (2005) [Association of genetic polymorphism in the DNA repair gene XRCC1 with susceptibility to lung cancer in non-smoking women]. Zhonghua Zhong Liu Za Zhi 27: 713-716.

181. Dufloth RM, Costa S, Schmitt F, Zeferino LC (2005) DNA repair gene polymorphisms and susceptibility to familial breast cancer in a group of patients from Campinas, Brazil. Genet Mol Res 4: 771-782.

182. Patel AV, Calle EE, Pavluck AL, Feigelson HS, Thun MJ, et al. (2005) A prospective study of XRCC1 (X-ray cross-complementing group 1) polymorphisms and breast cancer risk. Breast Cancer Res 7: R1168-R1173.

183. Pakakasama S, Sirirat T, Kanchanachumpol S, Udomsubpayakul U, Mahasirimongkol S, et al. (2007) Genetic polymorphisms and haplotypes of DNA repair genes in childhood acute lymphoblastic leukemia. Pediatr Blood Cancer 48: 16-20.

184. Duarte MC, Colombo J, Rossit AR, Caetano A, Borim AA, et al. (2005) Polymorphisms of DNA repair genes XRCC1 and XRCC3, interaction with environmental exposure and risk of chronic gastritis and gastric cancer. World J Gastroenterol 11: 6593-6600.

185. Chen L, Ambrosone CB, Lee J, Sellers TA, Pow-Sang J, et al. (2006) Association between polymorphisms in the DNA repair genes XRCC1 and APE1, and the risk of prostate cancer in white and black Americans. J Urol 175: 108-112, 112.

186. Kietthubthew S, Sriplung H, Au WW, Ishida T (2006) Polymorphism in DNA repair genes and oral squamous cell carcinoma in Thailand. Int J Hyg Environ Health 209: 21-29.

187. Zhai X, Liu J, Hu Z, Wang S, Qing J, et al. (2006) Polymorphisms of ADPRT Val762Ala and XRCC1 Arg399Glu and risk of breast cancer in Chinese women: a case control analysis. Oncol Rep 15: 247-252.

188. Ramachandran S, Ramadas K, Hariharan R, Rejnish KR, Radhakrishna PM (2006) Single nucleotide polymorphisms of DNA repair genes XRCC1 and XPD and its molecular mapping in Indian oral cancer. Oral Oncol 42: 350-362.

189. Brewster AM, Jorgensen TJ, Ruczinski I, Huang HY, Hoffman S, et al. (2006) Polymorphisms of the DNA repair genes XPD (Lys751Gln) and XRCC1 (Arg399Gln and Arg194Trp): relationship to breast cancer risk and familial predisposition to breast cancer. Breast Cancer Res Treat 95: 73-80.

190. Andrew AS, Nelson HH, Kelsey KT, Moore JH, Meng AC, et al. (2006) Concordance of multiple analytical approaches demonstrates a complex relationship between DNA repair gene SNPs, smoking and bladder cancer susceptibility. Carcinogenesis 27: 1030-1037.

191. Matullo G, Dunning AM, Guarrera S, Baynes C, Polidoro S, et al. (2006) DNA repair polymorphisms and cancer risk in non-smokers in a cohort study. Carcinogenesis 27: 997-1007.

192. Matullo G, Guarrera S, Sacerdote C, Polidoro S, Davico L, et al. (2005) Polymorphisms/haplotypes in DNA repair genes and smoking: a bladder cancer case-control study. Cancer Epidemiol Biomarkers Prev 14: 2569-2578.

193. Metsola K, Kataja V, Sillanpaa P, Siivola P, Heikinheimo L, et al. (2005) XRCC1 and XPD genetic polymorphisms, smoking and breast cancer risk in a Finnish case-control study. Breast Cancer Res 7: R987-R997.

194. Zienolddiny S, Campa D, Lind H, Ryberg D, Skaug V, et al. (2006) Polymorphisms of DNA repair genes and risk of non-small cell lung cancer. Carcinogenesis 27: 560-567.

195. Majumder M, Sikdar N, Paul RR, Roy B (2005) Increased risk of oral leukoplakia and cancer among mixed tobacco users carrying XRCC1 variant haplotypes and cancer among smokers carrying two risk genotypes: one on each of two loci, GSTM3 and XRCC1 (Codon 280). Cancer Epidemiol Biomarkers Prev 14: 2106-2112.

196. Chen CC, Yang SY, Liu CJ, Lin CL, Liaw YF, et al. (2005) Association of cytokine and DNA repair gene polymorphisms with hepatitis B-related hepatocellular carcinoma. Int J Epidemiol 34: 1310-1318.

197. Chan EC, Lam SY, Fu KH, Kwong YL (2005) Polymorphisms of the GSTM1, GSTP1, MPO, XRCC1, and NQO1 genes in Chinese patients with non-small cell lung cancers: relationship with aberrant promoter methylation of the CDKN2A and RARB genes. Cancer Genet Cytogenet 162: 10-20.

198. Schneider J, Classen V, Bernges U, Philipp M (2005) XRCC1 polymorphism and lung cancer risk in relation to tobacco smoking. Int J Mol Med 16: 709-716.

199. Zhu R, Lu FJ, Zhang ZB, Zhai XW, Liu J, et al. (2005) [Association of genetic polymorphism of XRCC1 with susceptibility to acute childhood leukemia]. Wei Sheng Yan Jiu 34: 300-302.

200. Yu MW, Yang SY, Pan IJ, Lin CL, Liu CJ, et al. (2003) Polymorphisms in XRCC1 and glutathione S-transferase genes and hepatitis B-related hepatocellular carcinoma. J Natl Cancer Inst 95: 1485-1488.

201. Ritchey JD, Huang WY, Chokkalingam AP, Gao YT, Deng J, et al. (2005) Genetic variants of DNA repair genes and prostate cancer: a population-based study. Cancer Epidemiol Biomarkers Prev 14: 1703-1709.

202. Niwa Y, Matsuo K, Ito H, Hirose K, Tajima K, et al. (2005) Association of XRCC1 Arg399Gln and OGG1 Ser326Cys polymorphisms with the risk of cervical cancer in Japanese subjects. Gynecol Oncol 99: 43-49.

203. Hu Z, Ma H, Lu D, Zhou J, Chen Y, et al. (2005) A promoter polymorphism (-77T>C) of DNA repair gene XRCC1 is associated with risk of lung cancer in relation to tobacco smoking. Pharmacogenet Genomics 15: 457-463.

204. Yeh CC, Hsieh LL, Tang R, Chang-Chieh CR, Sung FC (2005) MS-920: DNA repair gene polymorphisms, diet and colorectal cancer risk in Taiwan. Cancer Lett 224: 279-288.

205. Rydzanicz M, Wierzbicka M, Gajecka M, Szyfter W, Szyfter K (2005) The impact of genetic factors on the incidence of multiple primary tumors (MPT) of the head and neck. Cancer Lett 224: 263-278.

206. Festa F, Kumar R, Sanyal S, Unden B, Nordfors L, et al. (2005) Basal cell carcinoma and variants in genes coding for immune response, DNA repair, folate and iron metabolism. Mutat Res 574: 105-111.

207. Casson AG, Zheng Z, Evans SC, Veugelers PJ, Porter GA, et al. (2005) Polymorphisms in DNA repair genes in the molecular pathogenesis of esophageal (Barrett) adenocarcinoma. Carcinogenesis 26: 1536-1541.

208. Hung RJ, Brennan P, Canzian F, Szeszenia-Dabrowska N, Zaridze D, et al. (2005) Large-scale investigation of base excision repair genetic polymorphisms and lung cancer risk in a multicenter study. J Natl Cancer Inst 97: 567-576.

209. Shen M, Berndt SI, Rothman N, Mumford JL, He X, et al. (2005) Polymorphisms in the DNA base excision repair genes APEX1 and XRCC1 and lung cancer risk in Xuan Wei, China. Anticancer Res 25: 537-542.

210. Huang WY, Chow WH, Rothman N, Lissowska J, Llaca V, et al. (2005) Selected DNA repair polymorphisms and gastric cancer in Poland. Carcinogenesis 26: 1354-1359.

211. Hong YC, Lee KH, Kim WC, Choi SK, Woo ZH, et al. (2005) Polymorphisms of XRCC1 gene, alcohol consumption and colorectal cancer. Int J Cancer 116: 428-432.

212. Demokan S, Demir D, Suoglu Y, Kiyak E, Akar U, et al. (2005) Polymorphisms of the XRCC1 DNA repair gene in head and neck cancer. Pathol Oncol Res 11: 22-25.

213. Stern MC, Siegmund KD, Corral R, Haile RW (2005) XRCC1 and XRCC3 polymorphisms and their role as effect modifiers of unsaturated fatty acids and antioxidant intake on colorectal adenomas risk. Cancer Epidemiol Biomarkers Prev 14: 609-615.

214. Kirk GD, Turner PC, Gong Y, Lesi OA, Mendy M, et al. (2005) Hepatocellular carcinoma and polymorphisms in carcinogen-metabolizing and DNA repair enzymes in a population with aflatoxin exposure and hepatitis B virus endemicity. Cancer Epidemiol Biomarkers Prev 14: 373-379.

215. Shen J, Gammon MD, Terry MB, Wang L, Wang Q, et al. (2005) Polymorphisms in XRCC1 modify the association between polycyclic aromatic hydrocarbon-DNA adducts, cigarette smoking, dietary antioxidants, and breast cancer risk. Cancer Epidemiol Biomarkers Prev 14: 336-342.

216. Zhang X, Miao X, Liang G, Hao B, Wang Y, et al. (2005) Polymorphisms in DNA base excision repair genes ADPRT and XRCC1 and risk of lung cancer. Cancer Res 65: 722-726.

217. Yeh CC, Sung FC, Tang R, Chang-Chieh CR, Hsieh LL (2005) Polymorphisms of the XRCC1, XRCC3, & XPD genes, and colorectal cancer risk: a case-control study in Taiwan. BMC Cancer 5: 12.

218. Chacko P, Rajan B, Joseph T, Mathew BS, Pillai MR (2005) Polymorphisms in DNA repair gene XRCC1 and increased genetic susceptibility to breast cancer. Breast Cancer Res Treat 89: 15-21.

219. Deligezer U, Dalay N (2004) Association of the XRCC1 gene polymorphisms with cancer risk in Turkish breast cancer patients. Exp Mol Med 36: 572-575.

220. Gajecka M, Rydzanicz M, Jaskula-Sztul R, Wierzbicka M, Szyfter W, et al. (2005) Reduced DNA repair capacity in laryngeal cancer subjects. A comparison of phenotypic and genotypic results. Adv Otorhinolaryngol 62: 25-37.

221. Joseph T, Kusumakumary P, Chacko P, Abraham A, Pillai MR (2005) DNA repair gene XRCC1 polymorphisms in childhood acute lymphoblastic leukemia. Cancer Lett 217: 17-24.

222. Vogel U, Nexo BA, Wallin H, Overvad K, Tjonneland A, et al. (2004) No association between base excision repair gene polymorphisms and risk of lung cancer. Biochem Genet 42: 453-460.

223. Zhu QX, Bian JC, Shen Q, Jiang F, Tang HW, et al. (2004) [Genetic polymorphisms in X-ray repair cross-complementing gene 1 and susceptibility to papillary thyroid carcinoma]. Zhonghua Liu Xing Bing Xue Za Zhi 25: 702-705.

224. Liu G, Zhou W, Park S, Wang LI, Miller DP, et al. (2004) The SOD2 Val/Val genotype enhances the risk of nonsmall cell lung carcinoma by p53 and XRCC1 polymorphisms. Cancer 101: 2802-2808.

225. Ratnasinghe LD, Abnet C, Qiao YL, Modali R, Stolzenberg-Solomon R, et al. (2004) Polymorphisms of XRCC1 and risk of esophageal and gastric cardia cancer. Cancer Lett 216: 157-164.

226. Han J, Hankinson SE, Colditz GA, Hunter DJ (2004) Genetic variation in XRCC1, sun exposure, and risk of skin cancer. Br J Cancer 91: 1604-1609.

227. Krupa R, Blasiak J (2004) An association of polymorphism of DNA repair genes XRCC1 and XRCC3 with colorectal cancer. J Exp Clin Cancer Res 23: 285-294.

228. Popanda O, Schattenberg T, Phong CT, Butkiewicz D, Risch A, et al. (2004) Specific combinations of DNA repair gene variants and increased risk for non-small cell lung cancer. Carcinogenesis 25: 2433-2441.

229. Wang LE, Bondy ML, Shen H, El-Zein R, Aldape K, et al. (2004) Polymorphisms of DNA repair genes and risk of glioma. Cancer Res 64: 5560-5563.

230. Kelsey KT, Park S, Nelson HH, Karagas MR (2004) A population-based case-control study of the XRCC1 Arg399Gln polymorphism and susceptibility to bladder cancer. Cancer Epidemiol Biomarkers Prev 13: 1337-1341.

231. Tae K, Lee HS, Park BJ, Park CW, Kim KR, et al. (2004) Association of DNA repair gene XRCC1 polymorphisms with head and neck cancer in Korean population. Int J Cancer 111: 805-808.

232. Yu HP, Zhang XY, Wang XL, Shi LY, Li YY, et al. (2004) DNA repair gene XRCC1 polymorphisms, smoking, and esophageal cancer risk. Cancer Detect Prev 28: 194-199.

233. Hao B, Wang H, Zhou K, Li Y, Chen X, et al. (2004) Identification of genetic variants in base excision repair pathway and their associations with risk of esophageal squamous cell carcinoma. Cancer Res 64: 4378-4384.

234. Harms C, Salama SA, Sierra-Torres CH, Cajas-Salazar N, Au WW (2004) Polymorphisms in DNA repair genes, chromosome aberrations, and lung cancer. Environ Mol Mutagen 44: 74-82.

235. Matsuo K, Hamajima N, Suzuki R, Andoh M, Nakamura S, et al. (2004) Lack of association between DNA base excision repair gene XRCC1 Gln399Arg polymorphism and risk of malignant lymphoma in Japan. Cancer Genet Cytogenet 149: 77-80.

236. Figueiredo JC, Knight JA, Briollais L, Andrulis IL, Ozcelik H (2004) Polymorphisms XRCC1-R399Q and XRCC3-T241M and the risk of breast cancer at the Ontario site of the Breast Cancer Family Registry. Cancer Epidemiol Biomarkers Prev 13: 583-591.

237. Ito H, Matsuo K, Hamajima N, Mitsudomi T, Sugiura T, et al. (2004) Gene-environment interactions between the smoking habit and polymorphisms in the DNA repair genes, APE1 Asp148Glu and XRCC1 Arg399Gln, in Japanese lung cancer risk. Carcinogenesis 25: 1395-1401.

238. Forsti A, Angelini S, Festa F, Sanyal S, Zhang Z, et al. (2004) Single nucleotide polymorphisms in breast cancer. Oncol Rep 11: 917-922.

239. Rybicki BA, Conti DV, Moreira A, Cicek M, Casey G, et al. (2004) DNA repair gene XRCC1 and XPD polymorphisms and risk of prostate cancer. Cancer Epidemiol Biomarkers Prev 13: 23-29.

240. Shu XO, Cai Q, Gao YT, Wen W, Jin F, et al. (2003) A population-based case-control study of the Arg399Gln polymorphism in DNA repair gene XRCC1 and risk of breast cancer. Cancer Epidemiol Biomarkers Prev 12: 1462-1467.

241. Sanyal S, Festa F, Sakano S, Zhang Z, Steineck G, et al. (2004) Polymorphisms in DNA repair and metabolic genes in bladder cancer. Carcinogenesis 25: 729-734.

242. Shen M, Hung RJ, Brennan P, Malaveille C, Donato F, et al. (2003) Polymorphisms of the DNA repair genes XRCC1, XRCC3, XPD, interaction with environmental exposures, and bladder cancer risk in a case-control study in northern Italy. Cancer Epidemiol Biomarkers Prev 12: 1234-1240.

243. Smith TR, Levine EA, Perrier ND, Miller MS, Freimanis RI, et al. (2003) DNA-repair genetic polymorphisms and breast cancer risk. Cancer Epidemiol Biomarkers Prev 12: 1200-1204.

244. Moullan N, Cox DG, Angele S, Romestaing P, Gerard JP, et al. (2003) Polymorphisms in the DNA repair gene XRCC1, breast cancer risk, and response to radiotherapy. Cancer Epidemiol Biomarkers Prev 12: 1168-1174.

245. Matullo G, Guarrera S, Carturan S, Peluso M, Malaveille C, et al. (2001) DNA repair gene polymorphisms, bulky DNA adducts in white blood cells and bladder cancer in a case-control study. Int J Cancer 92: 562-567.

246. Abdel-Rahman SZ, Soliman AS, Bondy ML, Omar S, El-Badawy SA, et al. (2000) Inheritance of the 194Trp and the 399Gln variant alleles of the DNA repair gene XRCC1 are associated with increased risk of early-onset colorectal carcinoma in Egypt. Cancer Lett 159: 79-86.

247. Cho EY, Hildesheim A, Chen CJ, Hsu MM, Chen IH, et al. (2003) Nasopharyngeal carcinoma and genetic polymorphisms of DNA repair enzymes XRCC1 and hOGG1. Cancer Epidemiol Biomarkers Prev 12: 1100-1104.

248. Landi S, Gemignani F, Canzian F, Gaborieau V, Barale R, et al. (2006) DNA repair and cell cycle control genes and the risk of young-onset lung cancer. Cancer Res 66: 11062-11069.

249. Zhou W, Liu G, Miller DP, Thurston SW, Xu LL, et al. (2003) Polymorphisms in the DNA repair genes XRCC1 and ERCC2, smoking, and lung cancer risk. Cancer Epidemiol Biomarkers Prev 12: 359-365.

250. Misra RR, Ratnasinghe D, Tangrea JA, Virtamo J, Andersen MR, et al. (2003) Polymorphisms in the DNA repair genes XPD, XRCC1, XRCC3, and APE/ref-1, and the risk of lung cancer among male smokers in Finland. Cancer Lett 191: 171-178.

251. Smith TR, Miller MS, Lohman K, Lange EM, Case LD, et al. (2003) Polymorphisms of XRCC1 and XRCC3 genes and susceptibility to breast cancer. Cancer Lett 190: 183-190.

252. van Gils CH, Bostick RM, Stern MC, Taylor JA (2002) Differences in base excision repair capacity may modulate the effect of dietary antioxidant intake on prostate cancer risk: an example of polymorphisms in the XRCC1 gene. Cancer Epidemiol Biomarkers Prev 11: 1279-1284.

253. Sturgis EM, Castillo EJ, Li L, Zheng R, Eicher SA, et al. (1999) Polymorphisms of DNA repair gene XRCC1 in squamous cell carcinoma of the head and neck. Carcinogenesis 20: 2125-2129.

254. Seedhouse C, Bainton R, Lewis M, Harding A, Russell N, et al. (2002) The genotype distribution of the XRCC1 gene indicates a role for base excision repair in the development of therapy-related acute myeloblastic leukemia. Blood 100: 3761-3766.

255. Lee SG, Kim B, Choi J, Kim C, Lee I, et al. (2002) Genetic polymorphisms of XRCC1 and risk of gastric cancer. Cancer Lett 187: 53-60.

256. Li M, Yin Z, Cui Z, He Q, Zhou B (2005) [Association of genetic polymorphism in DNA repair gene XRCC1 with risk of lung adenocarcinoma in nonsmoking women]. Zhongguo Fei Ai Za Zhi 8: 431-434.

257. Duell EJ, Holly EA, Bracci PM, Wiencke JK, Kelsey KT (2002) A population-based study of the Arg399Gln polymorphism in X-ray repair cross- complementing group 1 (XRCC1) and risk of pancreatic adenocarcinoma. Cancer Res 62: 4630-4636.

258. Stern MC, Umbach DM, van Gils CH, Lunn RM, Taylor JA (2001) DNA repair gene XRCC1 polymorphisms, smoking, and bladder cancer risk. Cancer Epidemiol Biomarkers Prev 10: 125-131.

259. Chen S, Tang D, Xue K, Xu L, Ma G, et al. (2002) DNA repair gene XRCC1 and XPD polymorphisms and risk of lung cancer in a Chinese population. Carcinogenesis 23: 1321-1325.

260. Xing D, Qi J, Miao X, Lu W, Tan W, et al. (2002) Polymorphisms of DNA repair genes XRCC1 and XPD and their associations with risk of esophageal squamous cell carcinoma in a Chinese population. Int J Cancer 100: 600-605.

261. Kim SU, Park SK, Yoo KY, Yoon KS, Choi JY, et al. (2002) XRCC1 genetic polymorphism and breast cancer risk. Pharmacogenetics 12: 335-338.

262. Ratnasinghe D, Yao SX, Tangrea JA, Qiao YL, Andersen MR, et al. (2001) Polymorphisms of the DNA repair gene XRCC1 and lung cancer risk. Cancer Epidemiol Biomarkers Prev 10: 119-123.

263. Hu JJ, Smith TR, Miller MS, Lohman K, Case LD (2002) Genetic regulation of ionizing radiation sensitivity and breast cancer risk. Environ Mol Mutagen 39: 208-215.

264. Divine KK, Gilliland FD, Crowell RE, Stidley CA, Bocklage TJ, et al. (2001) The XRCC1 399 glutamine allele is a risk factor for adenocarcinoma of the lung. Mutat Res 461: 273-278.

265. Shen H, Xu Y, Qian Y, Yu R, Qin Y, et al. (2000) Polymorphisms of the DNA repair gene XRCC1 and risk of gastric cancer in a Chinese population. Int J Cancer 88: 601-606.

266. Olshan AF, Watson MA, Weissler MC, Bell DA (2002) XRCC1 polymorphisms and head and neck cancer. Cancer Lett 178: 181-186.

267. Park JY, Lee SY, Jeon HS, Bae NC, Chae SC, et al. (2002) Polymorphism of the DNA repair gene XRCC1 and risk of primary lung cancer. Cancer Epidemiol Biomarkers Prev 11: 23-27.

268. Nelson HH, Kelsey KT, Mott LA, Karagas MR (2002) The XRCC1 Arg399Gln polymorphism, sunburn, and non-melanoma skin cancer: evidence of gene-environment interaction. Cancer Res 62: 152-155.

269. David-Beabes GL, London SJ (2001) Genetic polymorphism of XRCC1 and lung cancer risk among African-Americans and Caucasians. Lung Cancer 34: 333-339.

270. Goncalves FT, Francisco G, de Souza SP, Luiz OC, Festa-Neto C, et al. (2011) European ancestry and polymorphisms in DNA repair genes modify the risk of melanoma: a case-control study in a high UV index region in Brazil. J Dermatol Sci 64: 59-66.

271. Wu X, Gu J, Grossman HB, Amos CI, Etzel C, et al. (2006) Bladder cancer predisposition: a multigenic approach to DNA-repair and cell-cycle-control genes. Am J Hum Genet 78: 464-479.

272. Broberg K, Bjork J, Paulsson K, Hoglund M, Albin M (2005) Constitutional short telomeres are strong genetic susceptibility markers for bladder cancer. Carcinogenesis 26: 1263-1271.

273. Zhi Y, Yu J, Liu Y, Wei Q, Yuan F, et al. (2012) Interaction between polymorphisms of DNA repair genes significantly modulated bladder cancer risk. Int J Med Sci 9: 498-505.

274. Thirumaran RK, Bermejo JL, Rudnai P, Gurzau E, Koppova K, et al. (2006) Single nucleotide polymorphisms in DNA repair genes and basal cell carcinoma of skin. Carcinogenesis 27: 1676-1681.

275. Winsey SL, Haldar NA, Marsh HP, Bunce M, Marshall SE, et al. (2000) A variant within the DNA repair gene XRCC3 is associated with the development of melanoma skin cancer. Cancer Res 60: 5612-5616.

276. Figl A, Scherer D, Nagore E, Bermejo JL, Botella-Estrada R, et al. (2010) Single-nucleotide polymorphisms in DNA-repair genes and cutaneous melanoma. Mutat Res 702: 8-16.

277. Huang WY, Olshan AF, Schwartz SM, Berndt SI, Chen C, et al. (2005) Selected genetic polymorphisms in MGMT, XRCC1, XPD, and XRCC3 and risk of head and neck cancer: a pooled analysis. Cancer Epidemiol Biomarkers Prev 14: 1747-1753.

278. Martinez-Balibrea E, Manzano JL, Martinez-Cardus A, Moran T, Cirauqui B, et al. (2007) Combined analysis of genetic polymorphisms in thymidylate synthase, uridine diphosphate glucoronosyltransferase and X-ray cross complementing factor 1 genes as a prognostic factor in advanced colorectal cancer patients treated with 5-fluorouracil plus oxaliplatin or irinotecan. Oncol Rep 17: 637-645.

279. HAMANO, MATSUI, OHTAKE, NAKATA, SUZUKI (2008) Polymorphisms of DNA repair genes, XRCC1 and XRCC3, and susceptibility to familial prostate cancer in a Japanese population. Asia-Pacific Journal of Clinical Oncology 4:21-26.

280. Laantri N, Jalbout M, Khyatti M, Ayoub WB, Dahmoul S, et al. (2011) XRCC1 and hOGG1 genes and risk of nasopharyngeal carcinoma in North African countries. Mol Carcinog 50: 732-737.

281. Yin J, Vogel U, Gerdes LU, Dybdahl M, Bolund L, et al. (2003) Twelve single nucleotide polymorphisms on chromosome 19q13.2-13.3: linkage disequilibria and associations with basal cell carcinoma in Danish psoriatic patients. Biochem Genet 41: 27-37.

282. Povey JE, Darakhshan F, Robertson K, Bisset Y, Mekky M, et al. (2007) DNA repair gene polymorphisms and genetic predisposition to cutaneous melanoma. Carcinogenesis 28: 1087-1093.

283. Yin J, Rockenbauer E, Hedayati M, Jacobsen NR, Vogel U, et al. (2002) Multiple single nucleotide polymorphisms on human chromosome 19q13.2-3 associate with risk of Basal cell carcinoma. Cancer Epidemiol Biomarkers Prev 11: 1449-1453.

284. Kiyohara C, Horiuchi T, Takayama K, Nakanishi Y (2012) Genetic polymorphisms involved in carcinogen metabolism and DNA repair and lung cancer risk in a Japanese population. J Thorac Oncol 7: 954-962.

285. Chang JS, Wrensch MR, Hansen HM, Sison JD, Aldrich MC, et al. (2009) Base excision repair genes and risk of lung cancer among San Francisco Bay Area Latinos and African-Americans. Carcinogenesis 30: 78-87.
