# Supplementary material for: Association between the XRCC1 Arg399Gln Polymorphism and Risk of Cancer: Evidence from 297 Case–Control Studies
Source: PLoS One. 2013 Oct 29;8(10):e78071. doi: 10.1371/journal.pone.0078071 (PMC3812151; doi:10.1371/journal.pone.0078071)
Supplement: Table S1 — Case-control studies included in the meta-analysis. (DOC) [file pone.0078071.s002.doc]

**Table S1** Case-control studies included in the meta-analysis

| First author/year | | | | | Country | Ethnicity | | | Case–control | | SC | Cancer type | HWE |
| --- | --- | --- | --- | --- | --- | --- | --- | --- | --- | --- | --- | --- | --- |
| Duell [80] 2001 | | | | USA | | African | | | 253–266 | | PB | Breast | Y |
| Duell [80] 2001 | | | | USA | | Caucasian | | | 386–381 | | PB | Breast | Y |
| Shu [240] 2003 | | | | China | | Asian | | | 1088–1182 | | PB | Breast | Y |
| Smith [243] 2003 | | | | USA | | Caucasian | | | 251–267 | | HB | Breast | Y |
| Moullan [244] 2003 | | | | France | | Caucasian | | | 254–312 | | PB | Breast | Y |
| Smith [251] 2003 | | | | USA | | Caucasian | | | 162–300 | | HB | Breast | Y |
| Han [73] 2003 | | | | USA | | Mixed | | | 986–1337 | | PB | Breast | Y |
| Deligezer [219] 2004 | | | | Turkey | | Caucasian | | | 151–133 | | HB | Breast | Y |
| Figueiredo [236] 2004 | | | | Canada | | Caucasian | | | 402–402 | | PB | Breast | Y |
| Försti [238] 2004 | | | | Finland | | Caucasian | | | 223–298 | | PB | Breast | Y |
| Dufloth [181] 2005 | | | | Brazil | | Mixed | | | 86–119 | | HB | Breast | Y |
| Patel [182] 2005 | | | | USA | | Mixed | | | 452–452 | | PB | Breast | Y |
| Chacko [218] 2005 | | | | India | | Asian | | | 123–123 | | HB | Breast | Y |
| Shen [215] 2005 | | | | USA | | Mixed | | | 1067–1110 | | PB | Breast | Y |
| Metsola [193] 2005 | | | | Finland | | Caucasian | | | 479–451 | | PB | Breast | Y |
| Zhai [187] 2006 | | | | China | | Asian | | | 302–639 | | HB | Breast | Y |
| Brewster [189] 2006 | | | | USA | | Mixed | | | 305–310 | | PB | Breast | Y |
| Zhang [178] 2006 | | | | USA | | Caucasian | | | 3039–2587 | | PB | Breast | Y |
| BCAC [161] 2006 ABCFS | | | | Australia | | Caucasian | | | 1472–828 | | PB | Breast | Y |
| BCAC [161] 2006 GENICA | | | | Germany | | Caucasian | | | 602–625 | | PB | Breast | Y |
| BCAC [161] 2006 IARC-Thai | | | | Thailand | | Asian | | | 460–388 | | HB | Breast | Y |
| BCAC [161] 2006 LSHTM | | | | UK | | Caucasian | | | 585–598 | | PB | Breast | Y |
| BCAC [161] 2006 Madrid | | | | Spain | | Caucasian | | | 808–770 | | HB | Breast | Y |
| BCAC [161] 2006 PBSC | | | | Poland | | Caucasian | | | 2699–2290 | | PB | Breast | Y |
| BCAC [161] 2006 US 3-state | | | | USA | | Caucasian | | | 1869–2587 | | PB | Breast | Y |
| BCAC [161] 2006 Seoul | | | | Korea | | Asian | | | 308–314 | | HB | Breast | Y |
| BCAC [161] 2006 USRTS | | | | USA | | Caucasian | | | 707–1051 | | PB | Breast | Y |
| Pachkowski [176] 2006 | | | | USA | | Caucasian | | | 1244–1122 | | PB | Breast | Y |
| Pachkowski [176] 2006 | | | | USA | | African | | | 761–676 | | PB | Breast | Y |
| Jin [163] 2006 | | | | China | | Asian | | | 83–251 | | PB | Breast | Y |
| Bu [172] 2006 | | | | USA | | Mixed | | | 190–180 | | HB | Breast | N |
| Thyagarajan [162] 2006 | | | | USA | | Mixed | | | 193–322 | | PB | Breast | Y |
| Silva [130] 2007 | | | | Portugal | | Caucasian | | | 241–456 | | HB | Breast | Y |
| Costa [159] 2007 | | | | Portugal | | Caucasian | | | 256–441 | | HB | Breast | Y |
| Sangrajrang [131] 2008 | | | | Thailand | | Asian | | | 507–424 | | HB | Breast | Y |
| Loizidou [125] 2008 | | | | Cyprus | | Caucasian | | | 1107–1176 | | PB | Breast | Y |
| Ali [120] 2008 | | | | USA | | Mixed | | | 40–48 | | HB | Breast | Y |
| Kipikasová [114] 2008 | | | | Slovak | | Caucasian | | | 114–113 | | HB | Breast | Y |
| Smith [107] 2008 | | | | USA | | Caucasian | | | 312–406 | | HB | Breast | Y |
| Smith [107] 2008 | | | | USA | | African | | | 52–74 | | HB | Breast | Y |
| Mitra [108] 2008 | | | | India | | Asian | | | 150–225 | | PB | Breast | Y |
| Saadat [127] 2008 | | | | Iran | | Caucasian | | | 186–187 | | PB | Breast | Y |
| Syamala [100] 2009 | | | | India | | Asian | | | 359–367 | | HB | Breast | N |
| Sobczuk [78] 2009 | | | | Poland | | Caucasian | | | 150–106 | | HB | Breast | Y |
| Sterpone [68] 2010 | | | | Italy | | Caucasian | | | 43–31 | | HB | Breast | Y |
| Ming-Shiean [66] 2010 | | | | China | | Asian | | | 395–531 | | HB | Breast | Y |
| Zipprich [60] 2010 | | | | USA | | Mixed | | | 525–624 | | FB | Breast | Y |
| Qian [59] 2010 | | | | China | | Asian | | | 666–789 | | PB | Breast | Y |
| Jelonek [54] 2010 | | | | Poland | | Caucasian | | | 104–551 | | PB | Breast | Y |
| Santos [31] 2010 | | | | Brazil | | Mixed | | | 65–85 | | PB | Breast | Y |
| Romanowicz [44] 2010 | | | | Poland | | Caucasian | | | 220–220 | | HB | Breast | Y |
| Liu [55] 2011 | | | | China | | Asian | | | 995–1004 | | HB | Breast | Y |
| Hussien [30] 2012 | | | | Egypt | | African | | | 100–100 | | HB | Breast | Y |
| Roberts [32] 2011 | | | | USA | | Mixed | | | 982–1770 | | PB | Breast | Y |
| Ratnasinghe [262] 2001 | | | China | | | | Asian | | 107–208 | | PB | Lung | Y |
| David-Beabes [269] 2001 | | | USA | | | | Caucasian | | 180–461 | | PB | Lung | Y |
| David-Beabes [269] 2001 | | | USA | | | | African | | 154–243 | | PB | Lung | Y |
| Divine [264] 2001 | | | USA | | | | Caucasian | | 172–143 | | HB | Lung | Y |
| Chen [259] 2002 | | | China | | | | Asian | | 103–99 | | PB | Lung | Y |
| Park [267] 2002 | | | Korea | | | | Asian | | 192–135 | | HB | Lung | Y |
| Misra [250] 2003 | | | USA | | | | Caucasian | | 315–313 | | PB | Lung | Y |
| Zhou [249] 2003 | | | USA | | | | Caucasian | | 1091–1240 | | PB | Lung | Y |
| Ito [237] 2004 | | | Japan | | | | Asian | | 178–448 | | HB | Lung | Y |
| Popanda [228] 2004 | | | German | | | | Caucasian | | 463–460 | | HB | Lung | Y |
| Harms [234] 2004 | | | German | | | | Caucasian | | 110–119 | | HB | Lung | Y |
| Zhang [216] 2005 | | | China | | | | Asian | | 1000–1000 | | PB | Lung | Y |
| Hung [208] 2005 | | | Multiple | | | | Caucasian | | 2049–2015 | | HB | Lung | Y |
| Vogel [222] 2004 | | | Danish | | | | Caucasian | | 256–269 | | PB | Lung | Y |
| Schneider [198] 2005 | | | German | | | | Caucasian | | 446–662 | | HB | Lung | Y |
| Shen [209] 2005 | | | China | | | | Asian | | 116–109 | | PB | Lung | Y |
| Chan [197] 2005 | | | China | | | | Asian | | 75–162 | | HB | Lung | Y |
| Hu [203] 2005 | | | China | | | | Asian | | 710–710 | | HB | Lung | Y |
| Zienolddiny [194] 2006 | | | Norway | | | | Caucasian | | 331–391 | | PB | Lung | Y |
| Matullo [191] 2006 | | | Multiple | | | | Caucasian | | 116–1094 | | PB | Lung | Y |
| Hao [168] 2006 | | | China | | | | Asian | | 1024–1118 | | PB | Lung | Y |
| Landi [248] 2006 | | | Multiple | | | | Caucasian | | 295–314 | | HB | Lung | Y |
| Ryk [160] 2006 | | | Sweden | | | | Caucasian | | 177–153 | | PB | Lung | Y |
| De Ruyck [142] 2007 | | | Belgium | | | | Caucasian | | 109–109 | | HB | Lung | Y |
| Pachouri [147] 2007 | | | India | | | | Asian | | 103–122 | | PB | Lung | Y |
| Yin [150] 2007 | | | China | | | | Asian | | 205–193 | | HB | Lung | Y |
| López-Cima [135] 2007 | | | Spain | | | | Caucasian | | 516–533 | | HB | Lung | Y |
| Improta [101] 2008 | | | Italy | | | | Caucasian | | 94–121 | | HB | Lung | Y |
| Li [115] 2008 | | | China | | | | Asian | | 350–350 | | HB | Lung | Y |
| Yin [99] 2009 | | | China | | | | Asian | | 45–52 | | HB | Lung | Y |
| Cote [95] 2009 | | | USA | | | | Caucasian | | 387–406 | | PB | Lung | Y |
| Cote [95] 2009 | | | USA | | | | African | | 115–121 | | PB | Lung | Y |
| Kim [50] 2010 | | | Korea | | | | Asian | | 139–217 | | HB | Lung | Y |
| Osawa [46] 2010 | | | Japan | | | | Asian | | 104–120 | | HB | Lung | Y |
| Qian [47] 2011 | | | China | | | | Asian | | 581–603 | | HB | Lung | Y |
| Janik [42] 2011 | | | Poland | | | | Caucasian | | 88–79 | | HB | Lung | Y |
| Li [28] 2011 | | | China | | | | Asian | | 455–443 | | HB | Lung | Y |
| Sreeja [129] 2008 | | | India | | | | Asian | | 211–211 | | HB | Lung | Y |
| Wang [8] 2012 | | | China | | | | Asian | | 209–256 | | HB | Lung | Y |
| Li [180] 2005 | | | China | | | | Asian | | 50–50 | | HB | Lung | Y |
| Kiyohara [284] 2012 | | | Japan | | | | Asian | | 462–379 | | HB | Lung | Y |
| Chang [285] 2009 | | | USA | | | | Caucasian | | 113–298 | | PB | Lung | Y |
| Chang [285] 2009 | | | USA | | | | African | | 255–280 | | PB | Lung | Y |
| Sanyal [241] 2004 | | | Swede | | | | Caucasian | | 311–246 | | HB | Bladder | Y |
| Mittal [119] 2008 | | | India | | | | Asian | | 140–190 | | HB | Bladder | Y |
| Shen [242] 2003 | | | Italy | | | | Caucasian | | 201–214 | | HB | Bladder | Y |
| Sak [146] 2007 | | | UK | | | | Caucasian | | 532–560 | | HB | Bladder | Y |
| Matullo [192] 2005 | | | Italy | | | | Caucasian | | 315–313 | | HB | Bladder | Y |
| Karahalil [154] 2006 | | | Turkey | | | | Caucasian | | 100–100 | | HB | Bladder | Y |
| Figueroa [155] 2007 | | | Spain | | | | Caucasian | | 1061–996 | | HB | Bladder | Y |
| Wu [271] 2006 | | | USA | | | | Caucasian | | 613–596 | | HB | Bladder | Y |
| Stern [258] 2001 | | | USA | | | | Caucasian | | 214–197 | | HB | Bladder | Y |
| Stern [258] 2001 | | | USA | | | | African | | 19–13 | | HB | Bladder | Y |
| Broberg [272] 2005 | | | Swede | | | | Caucasian | | 61–155 | | PB | Bladder | Y |
| Wang [65] 2010 | | | China | | | | Asian | | 234–253 | | HB | Bladder | Y |
| Andrew [132] 2008 | | | USA | | | | Caucasian | | 990–1253 | | HB | Bladder | Y |
| Zhi [273] 2012 | | | China | | | | Asian | | 311–302 | | HB | Bladder | Y |
| Arizono [123] 2008 | | | Japan | | | | Asian | | 251–251 | | HB | Bladder | Y |
| Kelsey [230] 2004 | | | USA | | | | Mixed | | 355–544 | | PB | Bladder | Y |
| Mittal [18] 2011 | | | India | | | | Asian | | 212–250 | | PB | Bladder | Y |
| Covolo [118] 2008 | | | Italy | | | | Caucasian | | 197–211 | | HB | Bladder | NA |
| Fontana [110] 2008 | | | France | | | | Caucasian | | 51–45 | | HB | Bladder | Y |
| Gao [57] 2010 | | | USA | | | | Caucasian | | 192–313 | | HB | Bladder | NA |
| Wen [86] 2009 | | | China | | | | Asian | | 160–582 | | HB | Bladder | N |
| Thirumaran [274] 2006 | | | Multiple | | | | Caucasian | | 529–533 | | HB | Skin | Y |
| Nelson [268] 2002 | | | USA | | | | Caucasian | | 745–431 | | PB | Skin | Y |
| Kang [149] 2007 | | | Korea | | | | Asian | | 209–205 | | HB | Skin | Y |
| Festa [206] 2005 | | | Multiple | | | | Caucasian | | 197–548 | | HB | Skin | Y |
| Han [226] 2004 | | | USA | | | | Mixed | | 204–815 | | PB | Skin | Y |
| Winsey [275] 2000 | | | UK | | | | Caucasian | | 125–211 | | HB | Skin | Y |
| Figl [276] 2010 | | | German | | | | Caucasian | | 1185–1271 | | HB | Skin | Y |
| Li [170] 2006 | | | USA | | | | Mixed | | 602–603 | | HB | Skin | Y |
| Chiyomaru [7] 2012 | | | Japan | | | | Asian | | 197–93 | | HB | Skin | N |
| Yin [281] 2003 | | | Denmark. | | | | Caucasian | | 20–20 | | HB | Skin | Y |
| Povey [282] 2007 | | | UK | | | | Caucasian | | 507–437 | | PB | Skin | Y |
| Yin [283] 2002 | | | USA | | | | Caucasian | | 63–97 | | HB | Skin | Y |
| Goncalves [270] 2011 | | | Brazil | | | | Mixed | | 199–207 | | HB | Skin | Y |
| Demokan [212] 2005 | | Turkey | | | | | Caucasian | | 95–98 | | HB | HNC | Y |
| Kietthubthew [186] 2006 | | Thailand | | | | | Asian | | 106–164 | | HB | HNC | Y |
| Akulevich [88] 2009 | | Multiple | | | | | Caucasian | | 255–595 | | HB | HNC | Y |
| Krupa [39] 2011 | | Poland | | | | | Caucasian | | 253–253 | | HB | HNC | Y |
| Cao [167] 2006 | | China | | | | | Asian | | 425–501 | | HB | HNC | Y |
| Kowalski [89] 2009 | | Poland | | | | | Caucasian | | 92–124 | | HB | HNC | Y |
| Chiang [105] 2008 | | China | | | | | Asian | | 283–469 | | HB | HNC | Y |
| Varzim [87] 2003 | | Portugal | | | | | Caucasian | | 88–178 | | HB | HNC | Y |
| Tae [231] 2004 | | Korea | | | | | Asian | | 129–157 | | HB | HNC | Y |
| Majumder [151] 2007 | | India | | | | | Asian | | 309–385 | | HB | HNC | Y |
| Li [139] 2007 | | USA | | | | | Caucasian | | 830–854 | | HB | HNC | Y |
| Applebaum [92] 2009 | | USA | | | | | Caucasian | | 483–547 | | PB | HNC | Y |
| Cho [247] 2003 | | China | | | | | Asian | | 334–282 | | HB | HNC | Y |
| Huang [277] 2005 | | USA | | | | | Caucasian | | 404–664 | | HB | HNC | Y |
| Huang [277] 2005 | | USA | | | | | African | | 121–93 | | HB | HNC | Y |
| Sturgis [253] 1999 | | USA | | | | | Mixed | | 203–424 | | HB | HNC | Y |
| Yang [137] 2007 | | China | | | | | Asian | | 153–168 | | PB | HNC | Y |
| Olshan [266] 2002 | | USA | | | | | Caucasian | | 98–161 | | HB | HNC | Y |
| Ramachandran [188] 2006 | | India | | | | | Asian | | 110–110 | | HB | HNC | Y |
| Ho [140] 2007 | | USA | | | | | Mixed | | 138–503 | | HB | HNC | Y |
| Kumar [21] 2012 | | India | | | | | Asian | | 278–278 | | HB | HNC | Y |
| Alsbeih [63] 2010 | | Saudi Arabia | | | | | African | | 60–50 | | HB | HNC | Y |
| Jelonek [54] 2010 | | Poland | | | | | Caucasian | | 104–251 | | PB | HNC | Y |
| Csejtei [79] 2009 | | Hungarian | | | | | Caucasian | | 108–102 | | HB | HNC | Y |
| Fard-Esfahani [19] 2011 | | Iran | | | | | Caucasian | | 155–190 | | HB | HNC | Y |
| Yang [116] 2008 | | China | | | | | Asian | | 72–72 | | HB | HNC | Y |
| Ho [94] 2009 | | USA | | | | | Mixed | | 251–503 | | HB | HNC | Y |
| García-Quispes [41] 2011 | | Spain | | | | | Caucasian | | 386–474 | | HB | HNC | Y |
| Gugatschka [40] 2011 | | Austria | | | | | Caucasian | | 168–463 | | PB | HNC | Y |
| Harth [111] 2008 | | German | | | | | Caucasian | | 310–300 | | HB | HNC | Y |
| Santos [3] 2012 | | Portugal | | | | | Caucasian | | 109–217 | | HB | HNC | Y |
| Kostrzewska-Poczekaj [9] 2012 | | Poland | | | | | Caucasian | | 290–158 | | HB | HNC | Y |
| Siraj [98] 2009 | | Saudi | | | | | ME | | 50–229 | | HB | HNC | Y |
| Gajecka [220] 2005 | | Poland | | | | | Caucasian | | 293–319 | | HB | HNC | Y |
| Rydzanicz [205] 2005 | | Poland | | | | | Caucasian | | 182–143 | | HB | HNC | Y |
| Ryu [24] 2011 | | Korea | | | | | Asian | | 111–100 | | HB | HNC | N |
| Zhu [223] 2004 | | China | | | | | Asian | | 105–105 | | HB | HNC | Y |
| Laantri [280] 2011 | | Multiple | | | | | African | | 512–477 | | HB | HNC | Y |
| Yeh [157] 2007 | | China | | | | | Asian | | 718–729 | | HB | Colorectal | Y |
| Sliwinski [104] 2008 | | Poland | | | | | Caucasian | | 100–100 | | HB | Colorectal | Y |
| Skjelbred [175] 2006 | | Norway | | | | | Caucasian | | 157–399 | | HB | Colorectal | Y |
| Jin [148] 2007 | | China | | | | | Asian | | 202–616 | | PB | Colorectal | Y |
| Krupa [227] 2004 | | Poland | | | | | Caucasian | | 51–100 | | HB | Colorectal | Y |
| Kasahara [103] 2008 | | Japan | | | | | Asian | | 68–121 | | HB | Colorectal | NA |
| Moreno [171] 2006 | | Spain | | | | | Caucasian | | 355–322 | | HB | Colorectal | Y |
| Improta [101] 2008 | | Italy | | | | | Caucasian | | 109–121 | | HB | Colorectal | Y |
| Curtin [74] 2009 | | Canada | | | | | Mixed | | 1582–1950 | | PB | Colorectal | Y |
| Hong [211] 2005 | | Korea | | | | | Asian | | 209–209 | | HB | Colorectal | Y |
| Martinez-Balibrea [278] 2007 | | Spain | | | | | Caucasian | | 70–82 | | HB | Colorectal | Y |
| Gil [35] 2012 | | Poland | | | | | Caucasian | | 133–100 | | HB | Colorectal | Y |
| Wang [64] 2010 | | India | | | | | Asian | | 302–291 | | HB | Colorectal | Y |
| Berndt [152] 2007 | | USA | | | | | Caucasian | | 692–708 | | PB | Colorectal | Y |
| Berndt [152] 2007 | | USA | | | | | Mixed | | 47–48 | | PB | Colorectal | Y |
| Stern [213] 2005 | | USA | | | | | Mixed | | 782–734 | | HB | Colorectal | Y |
| Stern [126] 2005 | | China | | | | | Asian | | 294–1120 | | PB | Colorectal | Y |
| Jelonek [54] 2010 | | Poland | | | | | Caucasian | | 113–295 | | PB | Colorectal | Y |
| Engin [20] 2011 | | Turkey | | | | | Caucasian | | 96–108 | | HB | Colorectal | Y |
| Canbay [34] 2011 | | Turkey | | | | | Caucasian | | 79–247 | | HB | Colorectal | Y |
| Zhao [13] 2012 | | China | | | | | Asian | | 486–970 | | HB | Colorectal | Y |
| Yin [12] 2012 | | Japan | | | | | Asian | | 685–776 | | PB | Colorectal | Y |
| Abdel-Rahman [246] 2000 | | Egypt | | | | | African | | 48–48 | | HB | Colorectal | Y |
| Muñiz-Mendoza [4] 2012 | | Mexico | | | | | Caucasian | | 103–120 | | HB | Colorectal | Y |
| Yeh [204] 2005 | | China | | | | | Asian | | 718–729 | | HB | Colorectal | Y |
| Gsur [33] 2011 | | Austria | | | | | Caucasian | | 85–1663 | | PB | Colorectal | Y |
| Brevik [49] 2010 | | USA | | | | | Mixed | | 305–360 | | FB | Colorectal | Y |
| Ye [173] 2006 | Sweden | | | | | | Caucasian | | 126–472 | | PB | Gastric | Y |
| Doecke [117] 2008 | Australia | | | | | | Mixed | | 303–1337 | | PB | Gastric | Y |
| Ratnasinghe [225] 2004 | USA | | | | | | Caucasian | | 86–418 | | PB | Gastric | Y |
| Shen [265] 2000 | China | | | | | | Asian | | 188–166 | | PB | Gastric | Y |
| Palli [53] 2010 | Italy | | | | | | Caucasian | | 289–546 | | PB | Gastric | Y |
| Huang [210] 2005 | Poland | | | | | | Caucasian | | 281–390 | | PB | Gastric | Y |
| Canbay [56] 2010 | Turkey | | | | | | Caucasian | | 40–247 | | HB | Gastric | Y |
| Duarte [184] 2005 | Brazil | | | | | | Mixed | | 160–150 | | HB | Gastric | Y |
| Yan [81] 2009 | China | | | | | | Asian | | 455–650 | | HB | Gastric | Y |
| Capellá [109] 2008 | Multiple | | | | | | Caucasian | | 245–1173 | | PB | Gastric | Y |
| Ruzzo [138] 2007 | Italy | | | | | | Caucasian | | 91–119 | | HB | Gastric | Y |
| Miao [164] 2006 | China | | | | | | Asian | | 500–1000 | | PB | Gastric | Y |
| Lee [255] 2002 | Korea | | | | | | Asian | | 190–172 | | HB | Gastric | Y |
| Engin [20] 2011 | Turkey | | | | | | Caucasian | | 94–108 | | HB | Gastric | Y |
| Chen [36] 2011 | China | | | | | | Asian | | 334–334 | | PB | Gastric | Y |
| Seedhouse [254] 2002 | UK | | | | | | Caucasian | | 133–178 | | HB | Leukemia | Y |
| Ganster [83] 2009 | Australia | | | | | | Caucasian | | 429–429 | | HB | Leukemia | Y |
| Matsuo [235] 2004 | Japan | | | | | | Asian | | 260–500 | | HB | Leukemia | Y |
| Batar [97] 2009 | Turkey | | | | | | Caucasian | | 70–75 | | HB | Leukemia | Y |
| Joseph [221] 2005 | India | | | | | | Asian | | 117–117 | | HB | Leukemia | Y |
| Abramenko [5] 2012 | Ukraine | | | | | | Caucasian | | 169–94 | | HB | Leukemia | Y |
| Pakakasama [183] 2007 | Thailand | | | | | | Asian | | 108–317 | | HB | Leukemia | Y |
| Canalle [38] 2011 | Brazil | | | | | | Caucasian | | 173–223 | | HB | Leukemia | Y |
| Canalle [38] 2011 | Brazil | | | | | | African | | 28–138 | | HB | Leukemia | Y |
| Duman [14] 2011 | Turkey | | | | | | Caucasian | | 73–50 | | HB | Leukemia | Y |
| Deligezer [136] 2007 | Turkey | | | | | | Caucasian | | 254–226 | | HB | Leukemia | Y |
| Stanczyk [62] 2011 | Poland | | | | | | Caucasian | | 97–131 | | HB | Leukemia | Y |
| Meza-Espinoza [71] 2009 | Mexico | | | | | | Caucasian | | 120–120 | | HB | Leukemia | Y |
| Tumer [61] 2010 | Turkey | | | | | | Caucasian | | 167–190 | | HB | Leukemia | Y |
| Zhu [199] 2005 | China | | | | | | Asian | | 63–66 | | HB | Leukemia | NA |
| Ritchey [201] 2005 | China | | | | | | Asian | 155–243 | | | PB | Prostate | Y |
| Xu [145] 2007 | China | | | | | | Asian | 207–235 | | | HB | Prostate | Y |
| van Gils [252] 2002 | USA | | | | | | Caucasian | 76–182 | | | PB | Prostate | Y |
| Rybicki [239] 2004 | USA | | | | | | Caucasian | 572–437 | | | FB | Prostate | Y |
| Rybicki [239] 2004 | USA | | | | | | Mixed | 65–43 | | | FB | Prostate | Y |
| Dhillon [76] 2011 | Australia | | | | | | Caucasian | 115–130 | | | HB | Prostate | Y |
| Hamano [279] 2008 | Japan | | | | | | Asian | 142–119 | | | HB | Prostate | Y |
| Zhang [75] 2010 | USA | | | | | | Caucasian | 190–195 | | | PB | Prostate | NA |
| Agalliu [77] 2010 | USA | | | | | | Caucasian | 1257–1240 | | | PB | Prostate | Y |
| Agalliu [77] 2010 | USA | | | | | | African | 144–82 | | | PB | Prostate | Y |
| Chen [185] 2006 | USA | | | | | | Caucasian | 228–217 | | | HB | Prostate | Y |
| Chen [185] 2006 | USA | | | | | | African | 96–115 | | | HB | Prostate | Y |
| Hirata [156] 2007 | Japan | | | | | | Asian | 165–165 | | | HB | Prostate | Y |
| Kuasne [52] 2011 | Brazil | | | | | | Mixed | 172–172 | | | HB | Prostate | Y |
| Mittal [18] 2011 | India | | | | | | Asian | 195–250 | | | PB | Prostate | Y |
| Berhane [29] 2012 | India | | | | | | Asian | 150–300 | | | HB | Prostate | Y |
| Gao [58] 2010 | USA | | | | | | Caucasian | 352–106 | | | HB | Prostate | Y |
| Mandal [69] 2010 | India | | | | | | Asian | 171–200 | | | HB | Prostate | N |
| Kirk [214] 2005 | Gambia | | | | | | African | | 149–194 | | HB | Hepatocellular | Y |
| Mohana [1] 2012 | India | | | | | | Asian | | 93–93 | | HB | Hepatocellular | Y |
| Gulnaz [2] 2012 | Pakistan | | | | | | Asian | | 74–125 | | HB | Hepatocellular | Y |
| Li [15] 2012 | China | | | | | | Asian | | 150–158 | | HB | Hepatocellular | Y |
| Kiran [93] 2009 | India | | | | | | Asian | | 63–289 | | HB | Hepatocellular | N |
| Pan [48] 2011 | China | | | | | | Asian | | 202–236 | | HB | Hepatocellular | Y |
| Long [165] 2006 | China | | | | | | Asian | | 257–649 | | HB | Hepatocellular | NA |
| Borentain [134] 2007 | France | | | | | | Caucasian | | 56–77 | | HB | Hepatocellular | Y |
| Yu [200] 2003 | China | | | | | | Asian | | 577–389 | | HB | Hepatocellular | Y |
| McWilliams [112] 2008 | USA | | | | | | Mixed | | | 473–612 | HB | Pancreatic | Y |
| Duell [257] 2002 | USA | | | | | | Caucasian | | | 250–832 | PB | Pancreatic | Y |
| Duell [257] 2002 | USA | | | | | | Asian | | | 17–51 | PB | Pancreatic | Y |
| Duell [257] 2002 | USA | | | | | | African | | | 26–36 | PB | Pancreatic | Y |
| Jiao [166] 2006 | USA | | | | | | Mixed | | | 380–354 | HB | Pancreatic | Y |
| Wang [158] 2006 | China | | | | | | Asian | | | 101–337 | HB | Pancreatic | Y |
| Lee [85] 2001 | China | | | | | | Asian | | | 105–264 | HB | Esophageal | Y |
| Liu [153] 2007 | USA | | | | | | Mixed | | | 183–336 | HB | Esophageal | Y |
| Casson [207] 2005 | Canada | | | | | | Caucasian | | | 56–95 | HB | Esophageal | Y |
| Ye [173] 2006 | Sweden | | | | | | Caucasian | | | 177–472 | PB | Esophageal | Y |
| Xing [260] 2002 | China | | | | | | Asian | | | 433–524 | PB | Esophageal | Y |
| Cai [169] 2006 | China | | | | | | Asian | | | 205–392 | PB | Esophageal | N |
| Doecke [117] 2008 | Australia | | | | | | Mixed | | | 263–1337 | PB | Esophageal | Y |
| Ferguson [121] 2008 | Ireland | | | | | | Caucasian | | | 209–248 | PB | Esophageal | Y |
| Hao [233] 2004 | China | | | | | | Asian | | | 411–479 | PB | Esophageal | Y |
| Sobti [141] 2007 | India | | | | | | Asian | | | 120–160 | HB | Esophageal | Y |
| Yu [232] 2004 | China | | | | | | Asian | | | 135–152 | HB | Esophageal | Y |
| Ratnasinghe [225] 2004 | USA | | | | | | Caucasian | | | 126–418 | PB | Esophageal | Y |
| Yu [27] 2011 | China | | | | | | Asian | | | 432–915 | PB | Esophageal | Y |
| Tse [113] 2008 | Canada | | | | | | Mixed | | | 311–452 | HB | Esophageal | Y |
| Niwa [202] 2005 | Japan | | | | | | Asian | | | 131–320 | PB | Cervical | Y |
| Settheetham-Ishida [26] 2011 | Thailand | | | | | | Asian | | | 111–118 | HB | Cervical | Y |
| Roszak [22] 2011 | Poland | | | | | | Caucasian | | | 189–308 | HB | Cervical | Y |
| Huang [143] 2007 | China | | | | | | Asian | | | 473–800 | HB | Cervical | Y |
| Barbisan [25] 2011 | Argentine | | | | | | Mixed | | | 103–114 | HB | Cervical | Y |
| Farkasova [6] 2008 | Slovakia | | | | | | Caucasian | | | 17–30 | HB | Cervical | NA |
| Baris [82] 2009 | Turkey | | | | | | Caucasian | | | 33–52 | HB | Lymphoma | Y |
| Liu [84] 2009 | China | | | | | | Asian | | | 221–254 | HB | Lymphoma | Y |
| Kim [72] 2010 | Korea | | | | | | Asian | | | 145–515 | HB | Lymphoma | Y |
| Smedby [179] 2006 | DS | | | | | | Caucasian | | | 428–593 | PB | Lymphoma | Y |
| Liu [96] 2009 | USA | | | | | | Caucasian | | | 373–364 | PB | Glioma | NA |
| Rajaraman [67] 2010 | USA | | | | | | Caucasian | | | 350–478 | HB | Glioma | Y |
| Felini [133] 2007 | USA | | | | | | Caucasian | | | 366–427 | PB | Glioma | Y |
| Yosunkaya [51] 2010 | Turkey | | | | | | Caucasian | | | 119–180 | HB | Glioma | Y |
| Zhou [10] 2011 | China | | | | | | Asian | | | 271–289 | HB | Glioma | Y |
| Wang [229] 2004 | USA | | | | | | Caucasian | | | 309–342 | HB | Glioma | Y |
| Kiuru [122] 2008 | Multiple | | | | | | Caucasian | | | 699–1549 | PB | Glioma | Y |
| Huang [128] 2008 | China | | | | | | Asian | | | 508–885 | PB | Biliary tract | Y |
| Hirata [177] 2006 | Japan | | | | | | Asian | | | 112–180 | HB | Renal cell | Y |
| Monroy [43] 2011 | USA | | | | | | Mixed | | | 199–219 | PB | Hodgkin | Y |
| Betti [45] 2011 | Italy | | | | | | Caucasian | | | 133–252 | PB | MM | Y |
| Srivastava [91] 2009 | India | | | | | | Asian | | | 173–204 | HB | Gallbladder | Y |
| Hsu [124] 2008 | China | | | | | | Asian | | | 210–218 | HB | UTC | Y |
| Bianchino [37] 2011 | Italy | | | | | | Caucasian | | | 290–242 | HB | UTC | Y |
| Sobczuk [11] 2012 | Poland | | | | | | Caucasian | | | 94–114 | HB | Endometrial | Y |
| Samulak [17] 2011 | Poland | | | | | | Caucasian | | | 456–300 | HB | Endometrial | Y |

DS Denmark and Sweden, ME Middle Eastern, MM malignant mesothelioma, UTC urinary transitional cell, PB population-based study, HB hospital-based study, FB family-based study, Y yes, N no, NA not available, HNC head and neck cancer, SC source of control, BCAC BCAC Breast Cancer Association Consortium, ABCFS Australian Breast Cancer Family Study, GENICA Gene Environment Interaction and Breast Cancer in Germany, IARC–Thai International Agency for Research on Cancer Breast Cancer Study in Thailand, LSHTM Breast Cancer Study & Mammography Oestrogens and Growth Factors Study, Madrid Spanish National Cancer Centre Breast Cancer Study, PBCS Polish Breast Cancer Study, US 3 state US Three-State Breast Cancer Study, Seoul Seoul Breast Cancer Study, USRTS US Radiologic Technologist Study
